# Graphene-Based Gas Sensors: State-of-the-Art Developments for Gas Sensing Applications

**DOI:** 10.3390/mi16080916

**Published:** 2025-08-08

**Authors:** Aviraj M. Teli, Sagar M. Mane, Sonali A. Beknalkar, Rajneesh Kumar Mishra, Wookhee Jeon, Jae Cheol Shin

**Affiliations:** 1 Division of Electronics and Electrical Engineering, Dongguk University-Seoul, Seoul 04620, Republic of Korea; avteli.teli@gmail.com (A.M.T.); sonaliabeknalkar@gmail.com (S.A.B.); 2 Department of Fiber System Engineering, Yeungnam University, 280 Dehak-Ro, Gyeongsan 38541, Gyeongbuk, Republic of Korea; manesagar99@gmail.com; 3 Department of Physics, Yeungnam University, Gyeongsan 38541, Gyeongbuk, Republic of Korea; 4 Department of Semiconductor, Convergence Engineering, Sungkyunkwan University, Suwon 16419, Gyeonggi, Republic of Korea; wookie92@skku.edu

**Keywords:** graphene, selectivity enhancement, environmental monitoring, industrial gas detection, gas sensing, sensor stability

## Abstract

Gas sensors based on graphene have gained considerable attention because of graphene’s remarkable properties, such as its extensive surface area, impressive electrical conductivity, and exceptional mechanical strength. This review critically analyzes recent developments in functionalization strategies designed to enhance the sensitivity, selectivity, and stability of graphene-based sensors. It discusses various chemical, physical, and hybrid functionalization methods, illustrating how surface alterations affect graphene’s interaction with target gas molecules. The paper also investigates the fundamental sensing mechanisms, including charge transfer, carrier mobility modulation, and Schottky barrier modification, to provide a thorough understanding of sensor response characteristics. Additionally, it highlights emerging applications in environmental monitoring, healthcare diagnostics, and industrial safety, demonstrating the transformative potential of these sensors in real-world settings. Finally, the review addresses challenges concerning reproducibility, long-term stability, and large-scale production, while also offering future insights on utilizing innovative nanomaterials and artificial intelligence to advance the next generation of graphene-based gas sensing technologies.

## 1. Introduction

In recent years, the rapid pace of industrialization has significantly impacted global economic growth and technological advancements [1,2,3,4]. Toxic gases in the atmosphere pose serious threats to both environmental and human health, causing respiratory issues, neurological damage, and chronic illnesses, which negatively impact both human and ecological well-being [5,6,7]. Furthermore, recent research suggests that specific volatile organic compounds in exhaled breath are associated with metabolic disorders, making breath analysis a valuable non-invasive diagnostic method that complements traditional blood and urine tests. This allows for early detection and real-time monitoring of various gases [8,9]. Consequently, gas sensors are designed to detect hazardous gases, playing a crucial role in environmental monitoring, industrial safety, and healthcare by providing real-time detection and enabling preventive actions against potentially life-threatening exposures [10,11]. Developing gas-sensing materials with ultrahigh surface-to-volume ratios and enhanced surface activity is vital for creating highly sensitive, low-temperature gas sensors. Their large specific surface areas promote strong gas-surface interactions, significantly improving sensitivity, response time, and overall sensing performance, making them ideal candidates for next-generation sensing technologies [12,13].

Graphene, a single atomic layer of sp^2^-hybridized carbon atoms arranged in a two-dimensional honeycomb lattice, continues to redefine the landscape of materials science due to its extraordinary combination of electrical, optical, thermal, and mechanical properties [14]. Since its discovery in 2004, graphene has emerged as a cornerstone material across multiple scientific domains, including electronics, photonics, energy storage, and biomedical devices [15,16]. Graphene’s primary attraction lies in its distinctive electronic structure, characterized by a linear dispersion relation at the Dirac point and zero-bandgap semimetallic nature, which results in massless charge carriers and exceptionally high electron mobility [17]. These characteristics have been pivotal in exploring relativistic quantum phenomena in solid-state physics, including Berry’s phase, the quantum Hall effect, and carrier transport that resembles massless Dirac fermions [18]. Moreover, its mechanical robustness provides stability, while flexibility enables the creation of wearable and bendable sensors. These features are further improved when graphene undergoes functionalization or is incorporated into hybrid composites, adding active sites and adjustable electronic qualities that enhance selective and sensitive gas detection. Additionally, graphene exhibits a wide-ranging optical response, featuring a universal absorbance in the visible spectrum and notable interactions with photons from ultraviolet to terahertz frequencies [19]. These optical properties have a significant impact on applications such as photodetectors, modulators, and transparent conductive films. In its functionalized form, such as graphene oxide (GO) and reduced graphene oxide (rGO), the optical and electronic properties of graphene can be further customized, offering adjustable band gaps and increased chemical reactivity [20]. Macroscopic graphene assemblies, such as fibers, films, and 3D networks, enable the realization of nanoscale benefits in practical materials, characterized by flexibility, high surface area, and strong electrical conductivity. This hierarchical structuring enhances graphene’s utility in various applications, ranging from supercapacitors to filtration membranes [21]. Graphene nanoribbons exhibit adjustable electronic and optical properties when exposed to external stimuli, such as electric and magnetic fields. Their edge states, quantum confinement, and Landau quantization result in complex spectra, thereby enhancing their suitability for optoelectronic and magneto-electronic applications [22]. With a theoretical Young’s modulus of about 1 TPa, thermal conductivity nearing 5000 W/m K, and intrinsic carrier mobility surpassing 200,000 cm^2^/V s, graphene has established itself as a leading nanomaterial for both fundamental research and practical applications [23]. Graphene’s unmatched surface area and chemical functionality also make it highly effective for environmental applications. Graphene-based nanomaterials are being actively investigated for use in water purification, sorption-based pollutant removal, and ecological sensing due to their ability to support catalytic and redox processes [24]. In sensor technologies, graphene’s two-dimensionality, electrical conductivity, and surface sensitivity enable highly responsive gas sensors. Through supramolecular engineering and the formation of composites with metal nanoparticles, graphene platforms have demonstrated remarkable selectivity and sensitivity to gases such as NO_2_ [25]. The synthesis of graphene and its related heterostructures or composites plays a pivotal role in tailoring their physicochemical properties for use in sensors, energy storage, and biomedical applications. Numerous scalable and controllable methods have been developed, each offering specific advantages and limitations. CVD remains one of the most widely used methods to produce high-quality, large-area graphene films. This method typically involves the decomposition of hydrocarbons at high temperatures on metal substrates, such as copper (Cu) or nickel (Ni), followed by transfer to the desired substrates. The post-synthesis treatment, including PMMA removal and annealing, has a significant impact on sensor performance by reducing impurities and enhancing surface homogeneity [26]. Furthermore, graphene oxide (GO), synthesized via the Hummers or modified Hummers method, is commonly used due to its ease of dispersion in aqueous media [27]. Reduction of GO into reduced graphene oxide (rGO) using agents like hydrazine, HI, or thermal treatment yields materials with tunable conductivity. However, a key challenge with reduced graphene oxide (rGO) is its tendency to restack caused by strong van der Waals forces between graphene sheets. This restacking reduces surface area and limits gas access. To address this, embedding graphene into composites with metal oxides, polymers, or carbon nanotubes has proven very effective. These materials act as spacers or disruptors, preserving interlayer gaps and increasing porosity. This structural separation enhances gas molecule access to active sites, improves electron transport, and boosts sensor sensitivity and response time. An advanced technique for synthesizing defect-rich graphene films involves molten salt treatment using NaNH_2_, with carbon nanodots acting as sacrificial spacers [28]. Upon pyrolysis, the CNDs prevent aggregation, enhance interlayer spacing, and create nanoporosity, thereby improving electrochemical performance without the need for additives. Additionally, hydrothermal and solvothermal solution-based techniques are useful for synthesizing graphene-based heterostructures, especially with metal oxides and polymers [29]. It allows the incorporation of materials into the graphene matrix and is suitable for constructing 3D architectures, including aerogels and foams. Moreover, a novel method for forming three-dimensional crumpled graphene nanosheets has been developed by supramolecular modification followed by lyophilisation [30]. This technique transforms 2D graphene oxide sheets into biomimetic 3D structures with an enhanced surface area and sensitivity, making them ideal for gas sensing. On the other hand, emerging methods such as screen printing, aerosol jet printing, and 3D printing of graphene/polymer inks enable the fabrication of wearable and flexible sensors [31,32]. These scalable approaches are compatible with industrial production of integrated devices. Interestingly, graphene is often composited with carbon nanotubes (CNTs), conducting polymers, or metal nanoparticles to enhance mechanical strength, flexibility, and selectivity. These composites are synthesized via in situ polymerization, electrophoretic deposition, or layer-by-layer assembly. Furthermore, doping graphene with heteroatoms such as B, N, S, and P introduces defect states, modulates electronic properties, and enhances analyte affinity, which is typically achieved during the thermal reduction of GO using dopant-rich precursors [33,34,35,36].

This review aims to provide a comprehensive and up-to-date overview of developments in graphene-based gas sensors. We begin by examining the essential properties of graphene that are pertinent to gas sensing. Next, we investigate various functionalization techniques, including covalent modifications, non-covalent interactions, doping, and the creation of hybrid nanocomposites, which are crucial for enhancing sensing performance. A comprehensive discussion of sensing mechanisms follows, clarifying the physical and chemical processes that underpin gas detection. Additionally, we emphasize the crucial performance metrics that determine sensor effectiveness and explore the latest emerging applications in environmental, healthcare, industrial, and wearable technologies. In addition, we confront existing challenges and future outlooks, pinpointing promising avenues for the ongoing development of graphene-based gas sensing platforms. This review aims to inspire novel innovations and collaborative efforts in the design, production, and use of advanced graphene-based gas sensors by critically assessing recent advancements and highlighting areas that require further exploration. Unlike earlier reviews that broadly examine material types or sensing results, this review emphasizes the relationship between functionalization and mechanisms in gas sensors. Additionally, this review explores emerging trends such as ternary hybrids, flexible electronics, and light-assisted sensing, which are set to revolutionize the field.

## 2. Structural Characterizations

X-ray diffraction (XRD) is an essential method for analyzing the structural properties of graphene and its derivatives. In graphite, the well-defined (002) peak at approximately 2θ ≈ 26.4° indicates an interlayer distance of about 3.35 Å, reflecting high crystallinity and regular stacking [37]. In contrast, few-layer graphene (FLG) exhibits a similar peak, but it is broader and less intense due to its fewer layers and stacking disorder. In single-layer graphene, the (002) peak is often extremely weak or absent, as the material lacks sufficient three-dimensional order to diffract X-rays effectively. Graphene oxide (GO) displays a pronounced shift in the main diffraction peak to around 2θ = 10–12°, reflecting increased interlayer spacing (~7–8 Å) caused by oxygen-containing functional groups [38]. Upon reduction to reduced graphene oxide (rGO), this peak diminishes or shifts back toward 24–26°, signifying partial restoration of graphitic order. The broadness and intensity of the XRD peaks serve as indicators of crystallite size, number of layers, and chemical modification. Raman spectroscopy is a widely used, non-destructive analytical technique for examining the structural and electronic properties of graphene. It provides a detailed view of the number of layers, disorder, doping, and functionalization. Its notable Raman features include the D band (~1350 cm^−1^), associated with graphene, the G band (~1580 cm^−1^), and the 2D band (~2700 cm^−1^) [39]. The G band is characterized by the E_2g_ phonon mode of bonded carbon atoms in graphene and is typical of graphitic materials. The D band is defect-activated, appearing as the breathing modes of six-atom rings, and requires defects for its activation. Therefore, the I_D_/I_G_ intensity ratio is frequently used to evaluate the level of structural disorder or defect density in graphene. The 2D band, in particular, indicates the number of graphene layers. In monolayer graphene, a sharp, symmetric, and usually high-intensity 2D peak is observed (I_2D_/I_G_ > 2) [40]. As more layers are added, the 2D band broadens and shifts the Raman band position. Its intensity decreases because of interlayer coupling and changes in the electronic band structure. Consequently, the 2D band serves as a reliable fingerprint to distinguish between single-layer, few-layer, and multilayer graphene. A prominent D band appears in the Raman spectrum of graphene oxide (GO) due to the presence of oxygen-containing groups, which introduce sp^3^-hybridized carbon atoms. The I_D_/I_G_ ratio increases significantly, indicating a high level of chaos. During chemical or thermal reduction to produce reduced graphene oxide (rGO), the sp^2^ network is not completely reconstructed, even though the I_D_/I_G_ ratio often remains high due to the formation of new defects during the process [41]. Fourier transform-infrared (FTIR) spectroscopy is a crucial technique for identifying functional groups and analyzing the chemical composition of graphene-based materials, particularly in their functionalized and oxidized forms, such as graphene oxide (GO) and reduced graphene oxide (rGO). While pure graphene exhibits weak infrared signals because of its symmetric, sp^2^-bonded carbon structure and absence of polar groups, FTIR proves to be highly effective when the structure is functionalized with specific chemical groups. For graphene oxide (GO), the FTIR spectrum typically exhibits several key absorption bands associated with oxygen-containing groups. The broad peak between 3200 and 3500 cm^−1^ corresponds to O-H stretching vibrations from hydroxyl groups and water molecules on the surface [42]. A strong band near 1720 cm^−1^ indicates C=O stretching from carbonyl or carboxylic acid groups. Additionally, a peak around 1620 cm^−1^ is linked to C=C skeletal vibrations in unoxidized sp^2^ regions, while bands at 1220–1260 cm^−1^ and 1040–1100 cm^−1^ are associated with C-O-C and C-O stretching, respectively [43]. These features verify extensive oxidation and the breakdown of the aromatic framework in GO. In the case of reduced graphene oxide (rGO), many oxygen-related bands decrease or vanish, indicating partial restoration of the graphene lattice and removal of oxygen functionalities. The peaks for OH and C=O decrease as the C=C peak grows stronger, reflecting the reformation of sp^2^ carbon [44]. X-ray Photoelectron Spectroscopy (XPS) is a crucial surface analysis method often used to examine the elemental composition, chemical states, and bonding environments in graphene and its derivatives. It is particularly valuable for characterizing functionalized forms, such as graphene oxide (GO) and reduced graphene oxide (rGO), by providing details on the presence and amount of oxygen-containing functional groups on the graphene surface. The most prominent feature in the XPS spectrum of graphene-based materials is the C1s core-level peak, which provides detailed information about various carbon bonding configurations. In pristine graphene, the C1s peak is located around 284.4 eV, corresponding to sp^2^-hybridized C=C bonds [45]. However, in graphene oxide (GO), the C1s spectrum becomes more complex, displaying multiple components due to oxidized carbon species. These typically include peaks for C-O (hydroxyl/epoxy) at approximately 286.7 eV, C=O (carbonyl) at about 287.6 eV, and O-C=O (carboxyl) at roughly 288.5–289.0 eV [46]. The relative intensities of these peaks indicate the degree of oxidation and disruption of the π-conjugated carbon network. After chemical or thermal reduction of GO to rGO, XPS shows a dramatic reduction, associated with a decrease in the intensity of all oxygen-related signals. At the same time, a relative increase is observed in the sp^2^ carbon peak at 284.4 eV. This indicates a partial restoration of the graphitic structure and the eviction of oxygen functionalities. Besides carbon, the O1s spectrum provides information on the complementary oxygen species. Reduction of the O/C atomic ratio is another evidence of successful deoxygenation.

**Figure 1:**
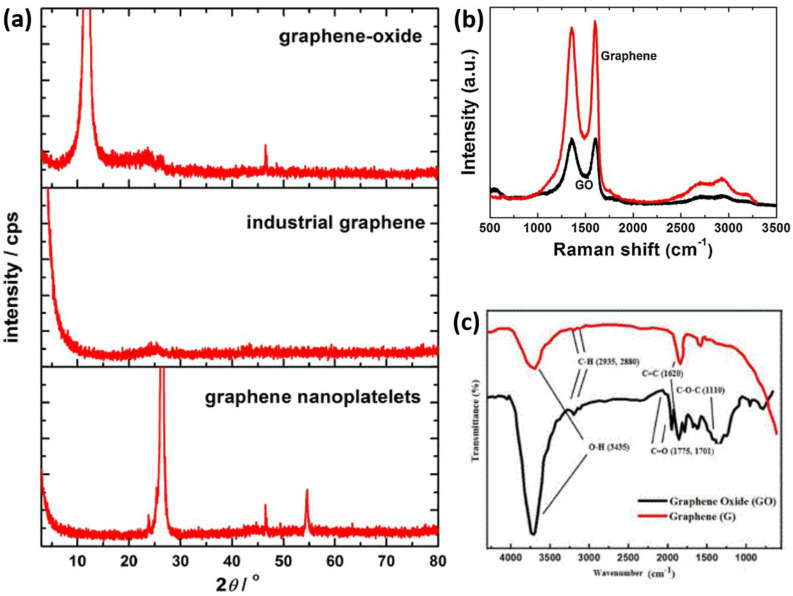
(**a**) XRD spectra of the graphene oxide (GO), industrial graphene, and graphene nanoplatelets. Reproduced with permission from Ref. [47]. Copyright (2016) Elsevier. (**b**) Raman vibrational spectra of the graphene and GO. Reproduced with permission from Ref. [48]. Copyright (2014) Elsevier. (**c**) FTIR spectra of the graphene and GO. Reproduced with permission from Ref. [49]. Copyright (2020) Nature Publishing Group.

Figure 1a illustrates the X-ray diffraction (XRD) schematic of three types of graphene, such as graphene oxide (GO), industrial graphene, and graphene nanoplatelets, highlighting their diverse structural features [47]. GO exhibits a strong peak at 2θ = 11.8°, characteristic of an increased interlayer spacing (~0.8 nm) due to the presence of numerous oxygen functional groups. Conversely, the strong (002) and (004) reflections at 2θ = 26.4° and 54.4°, respectively, exhibited by graphene nanoplatelets are characteristic of highly ordered graphite with minimal surface oxidation and extremely high conductivity. On the other hand, industrial graphene exhibits a broad (002) peak at 2θ = 25°, indicating partial disorder coupled with a modest degree of exfoliation. Figure 1b compares the Raman spectra of graphene oxide (GO) and hydrothermally reduced graphene, which inherently contains structural defects and does not display the symmetric, sharp 2D band characteristic of pristine monolayer graphene [48]. Raman spectra showed a large D band at 1353 cm^−1^, associated with disordered carbon due to oxygen functional groups, and a G band at 1605 cm^−1^, which is specific to the vibration of carbon atoms in a plane. Additionally, the G band of graphene exhibited weak downshifting to 1600 cm^−1^, which supports the reduction process. A wider 2D band at around 2700 cm^−1^ further confirmed the formation of few-layer graphene, whereas the formation of the S3 band at 2900 cm^−1^ indicated better graphitization and lack of defects. Therefore, Raman result evidence demonstrates that the hydrothermal method effectively produces graphene with greater structural order and fewer defects, supporting large-scale production. The FTIR spectrum of pristine graphene and graphene oxide (GO) is depicted in Figure 1c, illustrating the effective functionalization of graphene by the acidic-oxidation method [49]. A strong and broad absorption band at ~3435 cm^−1^ present in the FTIR spectrum of GO is assigned to the O-H stretch of hydroxyl groups. Although a similar peak is evident in pristine graphene, its intensity is extremely low, reflecting the microscopic presence of oxygenated functionalities. There are 1775 cm^−1^ and 1701 cm^−1^ peaks observed in GO, but not in pristine graphene, attributed to the C=O stretching vibrations of the carbonyl and carboxylic acid functional groups. The reduction between ~1740 cm^−1^ and ~1110 cm^−1^, followed by a peak at ~1110 cm^−1^, is associated with C-O-C stretching and represents the presence of epoxy or alkoxy functional groups, which form during oxidation. Both spectra exhibit a peak at ~1620 cm^−1^, corresponding to the C=C stretching vibration of the sp^2^ carbon structure, indicating that the fundamental graphene structure remains intact despite oxidative conversion. This preservation is crucial because it maintains the conductive and mechanical characteristics of the graphene sheets, which are believed to be essential for their use in sensing applications.

**Figure 2:**
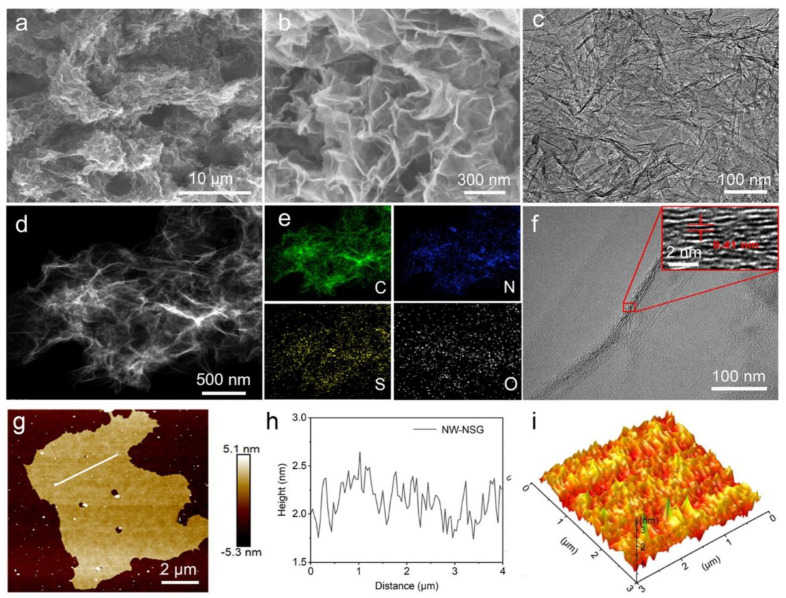
Morphological and structural characterization of nanowrinkle-enriched nitrogen and sulfur co-doped graphene (NW-NSG). (**a**,**b**) SEM images showing uniformly distributed MPP polymer coatings and contraction-induced nanoscale wrinkles on graphene sheets, (**c**) TEM image highlighting the furry-like MPP coating on graphene, (**d**) HAADF-STEM image and (**e**) corresponding EDS elemental mapping confirming homogeneous distribution of C, N, S, and O, (**f**) HRTEM image displaying enlarged interlayer spacing (~0.41 nm), indicative of heteroatom doping and nanowrinkling, (**g**) AFM topographic image and (**h**) its corresponding height profile, demonstrating nanoscale surface features, and (**i**) 3D AFM visualization revealing well-defined, sharp wrinkles across the NW-NSG surface. Reproduced with permission from Ref. [50]. Copyright (2025) American Chemical Society.

Graphene’s morphological and structural features can be effectively characterized using electron microscopy techniques, such as SEM, TEM, and HRTEM. These methods reveal a single-atom carbon lattice arranged in a hexagonal honeycomb pattern. All imaging techniques offer essential insights into graphene’s morphology, which directly affect its performance in various applications, particularly gas sensing. Wrinkled structures of graphene are particularly beneficial for gas sensing, as they provide a larger surface area for gas interaction, leading to an enhanced sensor response. It is well-documented that the morphology of graphene significantly influences its gas sensing capabilities. Factors such as surface roughness, defect density, and edge structure are known to influence the interaction of gas molecules with the surface, thereby affecting sensor properties like sensitivity, selectivity, and response time. For instance, defects and wrinkles increase the number of adsorption sites, enhancing sensor responses. Additionally, controlled defect levels and uniform monolayer graphene films can achieve high sensitivity due to reduced electronic noise and a large surface-to-volume ratio. Insights from morphology studies using SEM, TEM, and HRTEM enable researchers to design graphene-based gas sensors with surface characteristics tailored for specific gases. Figure 2a–i comprehensively illustrates the morphology, composition, and nanoscale features of the nanowrinkle-enriched nitrogen and sulfur co-doped graphene (NW-NSG), which is engineered through a polymer-contraction strategy [50]. The SEM images reveal a crumpled, textured surface where the polymer coating (melamine polyphosphate, MPP) induces nanoscale wrinkles across the graphene sheets, as illustrated in Figure 2a,b. The TEM image in Figure 2c further confirms the presence of a dense MPP layer with a “furry-like” appearance, indicating successful coating and morphological modification. Figure 2d,e shows the HAADF-STEM image and the accompanying EDS elemental maps, which confirm the homogeneous distribution of key heteroatoms (N, S, O). The high-resolution TEM image in Figure 2f shows an interlayer spacing of approximately 0.41 nm, wider than pristine graphene’s (~0.34 nm), which can be attributed to the induced wrinkling and doping. Figure 2g,h illustrates the AFM and height profiles of NW-NSG, providing quantitative evidence of the topological features and showcasing the nanowrinkle morphology and increased surface roughness. Figure 2i elucidates a 3D AFM image that visually emphasizes the uniform and sharp nanowrinkle topology.

## 3. Gas Sensing Parameters

These key sensing parameters are vital for assessing gas sensor performance. They affect application suitability as well as material design and optimization. Essential factors include sensitivity, selectivity, response and recovery times, detection limit, stability, reproducibility, and operating temperature.

### 3.1. Sensitivity

Sensitivity is a fundamental parameter in gas sensor performance, reflecting a sensor’s ability to detect small changes in the concentration of the target gas [51]. Typically, it is defined as the ratio of the change in an electrical signal, such as resistance, voltage, or current, to the change in gas concentration. The importance of high sensitivity lies in the fact that many hazardous or biologically relevant gases exist at trace levels, where even minor increases can have significant health, safety, or environmental impacts. Therefore, sensors must produce reliable and usable signals even at very low gas concentrations. Interestingly, sensitivity is more than just a feature; it is a fundamental requirement of any modern gas sensor. It underpins the success of various sensors used in areas like industrial safety, environmental protection, medical diagnostics, agriculture, and national security. As sensor technology advances, becoming more miniaturized, portable, and integrated into intelligent systems, the focus remains on increasing sensitivity. This is achieved through the use of advanced materials and innovative sensor structures, making sensitivity a central theme in ongoing research.

### 3.2. Selectivity

Selectivity is a key parameter of a gas sensor, reflecting its capacity to differentiate the target gas from other interfering substances in a mixture [52]. In real-world applications, such as industrial emissions, environmental monitoring, medical diagnostics, or safety systems, gases seldom exist in isolation. Thus, accurately detecting and measuring specific gases is crucial to prevent false readings and ensure dependable results. For instance, when detecting ammonia in human breath or hydrogen sulfide at industrial sites, cross-sensitivity issues may arise unless the sensor has robust selectivity features in its detection process. The importance of selectivity applies across different gas sensing performances. The high selectivity of gases reduces the chance of false gas response and enhances the accuracy of the sensing system in a mixed-gas environment. It is also capable of detecting at very low limits, such as ppm or even ppb levels, which is crucial for disease diagnosis, air monitoring, and early detection of gas leaks. Additionally, by selectively interacting with target molecules, the sensor’s durability, lifespan, and accuracy are maintained through the minimization of degradation of the sensing material. Achieving high selectivity remains a significant challenge in gas sensor technology. Advances in discovering new materials, engineering their surface modifications, and data analysis have significantly enhanced our ability to distinguish gases accurately. As gas sensing technology advances toward more complex, real-time, and portable devices, selectivity remains essential for developing intelligent and reliable sensors tailored to specific applications.

### 3.3. Response and Recovery Time

Response and recovery times are key parameters that influence the real-time performance and effectiveness of gas sensors [53]. Response time measures how long it takes for a sensor to reach 90% of its maximum signal after encountering the target gas. Recovery time refers to the period during which the sensor returns to its baseline signal after the gas is removed. These two parameters, together, characterize the sensor’s dynamic response and are critical for applications requiring the quick detection and clearance of gases, such as environmental monitoring, industrial safety, homeland security, and healthcare diagnostics. Real-time monitoring, quick response, and recovery are essential factors. In situations like toxic gas leaks, breathing diagnostics, or medical evaluations, any delay in detecting gas signals can lead to inaccurate results, missed warnings, or harm. Improving the sensor’s response dynamics enhances both accuracy and safety, making it more dependable. Response and recovery times are crucial factors in evaluating the performance of gas sensors. Improving these times is vital for reliable real-time detection in various gas applications. This enhancement is achieved by incorporating advanced nanomaterials such as graphene, which is expected to usher in a new era of gas sensors due to their superior dynamic performance over traditional, slower sensors.

### 3.4. Detection Limit

The limit of detection (LOD) of a gas sensor refers to the lowest concentration of a target gas that it can identify above the background noise [54]. This key parameter is crucial for evaluating a sensor’s performance, as it influences how effectively it can operate in different settings, including industrial, environmental, and biomedical fields. A lower detection limit signifies higher sensitivity, enabling the sensor to detect trace amounts of gases that might otherwise go unnoticed. This capability is especially vital in early detection scenarios, where small concentrations of toxic, greenhouse, or disease-related gases can have a significant impact on safety, diagnosis, or environmental health. Consequently, achieving a low detection limit is both a technical milestone and a practical requirement for enhancing gas sensing across various disciplines.

### 3.5. Stability

Stability is a crucial parameter for evaluating gas sensors, particularly in applications that require long-term reliability [55]. It reflects the sensor’s ability to maintain consistent sensitivity, selectivity, and baseline signals without sudden drifting, deterioration, or loss of function. Poor stability can lead to inaccurate measurements, false positives or negatives, and reduced sensor lifespan, potentially compromising systems that rely on continuous environmental or industrial monitoring. Therefore, stability is essential for the effective use of gas sensors in air quality assessment, industrial safety, automotive emissions control, and medical diagnostics. Gas sensors are susceptible to environmental and inherent factors that can significantly impact their stability. External influences, such as humidity, temperature fluctuations, and exposure to corrosive gases, can cause physical and chemical degradation of the sensing materials. Additionally, inherent factors such as aging, surface poisoning, and structural erosion contribute to performance drift over time.

### 3.6. Reproducibility

A key performance benchmark for gas sensors during testing and operation is reproducibility [56]. This refers to a sensor’s ability to produce consistent and reliable responses when repeatedly exposed to the same target gas concentration under identical conditions. Reproducibility is essential because it ensures that data remains accurate over time, allowing for confident decision-making. It is especially critical in commercial and industrial use, where it directly influences the credibility and dependability of monitoring systems. Without reproducibility, sensor readings can become inconsistent, leading to unpredictable variations. Reproducibility in gas sensor development depends on several key factors, including the consistency of the sensing material, fabrication accuracy, environmental stability, and resistance to degradation over time. For example, nanostructured metal oxides and functionalized 2D materials have shown promise due to their large surface area and high stability under operating conditions. Reproducible sensors exhibit minimal signal drift and variability. Reproducibility’s importance extends beyond performance; therefore, it is essential for sensor standardization, gaining regulatory approval, and achieving market acceptance. Industries like food safety, healthcare, and environmental protection require consistent sensor outputs to adhere to compliance and quality standards. Additionally, reproducible sensors support cross-laboratory comparisons, facilitate data sharing, and enable integration into larger sensor networks. In research and development, high reproducibility ensures that experimental results are reliable and can be duplicated by others, fostering scientific integrity and encouraging innovation in sensor technology.

### 3.7. Operating Temperature

The operating temperature of gas sensors is a crucial factor that influences their performance and design, impacting sensitivity, selectivity, response time, and recovery time [57]. Typically, conventional gas sensors operate at temperatures between 200 °C and 400 °C to achieve optimal reaction responses. Higher operating temperatures favor surface reactions between target gas molecules and the sensing material, enhancing charge transfer and improving sensing capabilities. However, operating at such high temperatures significantly increases power consumption, limiting the portability and integration of these sensors into compact, low-power, or wearable devices. Notably, graphene and other 2D materials have revolutionized gas sensor technology, particularly in terms of operating temperatures. Their high surface area, exceptional electrical conductivity, and easily modifiable defect sites or functional groups enable sensors made from waste-derived materials to be active at room temperature. Operating at room temperature not only reduces power consumption but also minimizes thermal stress on sensor components, resulting in longer-lasting and more reliable devices. Consequently, graphene-based sensors are particularly promising for environmental monitoring, medical diagnostics, and the Internet of Things (IoT), where low power use and compact size are essential.

## 4. Functionalization Strategies: Material Design and Sensing Implications

### 4.1. NH_3_ Gas Sensing

Due to its high reactivity and unpleasant odor, ammonia (NH_3_) is widely utilized in agriculture, the chemical industry, refrigeration, and waste treatment [58]. Because it is corrosive and toxic at any level, consistent monitoring for NH_3_ is essential for environmental protection, workplace safety, and public health [59]. To address these challenges, recent progress in NH_3_ sensing has utilized graphene and its composites or heterostructures, emphasizing important innovations in materials, sensing mechanisms, performance metrics, and the possibilities for both commercial and environmental applications. Aligayev et al. investigate the adsorption of ammonia (NH_3_) and methane (CH_4_) on Ni-doped graphene using both DFT and SCC-DFTB methods [60]. The introduction of Ni significantly impacts graphene’s electrons, enhancing interactions with gas molecules. With the addition of Ni, CH_4_ breaks down into CH_3_ and H, while NH_3_ decomposes into NH_2_ and H, resulting in the production of N_2_H_4_. The findings indicate that NH_3_ has a stronger interaction with the catalyst compared to CH_4_, and it also dissociates more readily. It was observed that Ni-doped graphene can sense more effectively, as evidenced by charge transfer, a change in optical absorbance, and improved electron movement. Additionally, the lower activation energies for Ni-doped graphene imply better gas sensing performance and greater stability. This indicates that doping graphene with Ni enhances its gas sensing performance, making it significantly more effective than regular graphene in applications like hydrogen production and gas sensing, thus advancing the development of innovative 2D materials. Similarly, a nanocomposite made of poly(methyl methacrylate), reduced graphene oxide, and zinc oxide (PRZ) significantly improved the effectiveness of room-temperature gas sensors [61]. Notable features include a 527% response rate at 350 ppm, swift response (1.94 s) and recovery times (13.02 s), and stability exceeding 90 days with less than 3.2% variation. The combination of RGO and ZnO nanoparticles within the PMMA matrix enhances the material’s conductivity and provides a greater surface area for gas adsorption. Additionally, the presence of n-p heterojunctions in the PRZ structure enhances the sensor’s ability to detect specific gases consistently. This demonstrates that the PRZ nanocomposite serves as a practical, energy-efficient, and durable solution for ammonia sensing in monitoring environments. In addition, an all-optical gas sensor was designed for NH_3_ sensing, utilizing a light-transmitting hybrid waveguide architecture composed of graphene ribbons and microfibers (GMHW) [62]. NH_3_ gas influences the operation of graphene as a gas sensor, altering the GMHW’s effective refractive index. These changes are detected through wavelength shifts in a Mach-Zehnder interferometer (MZI). The GMHW sensor exhibits a sensitivity of approximately 6 pm/ppm with a measurement resolution of 0.3 ppm. GMHW sensors react quickly (around 0.4 s) and can return to their original state, outperforming conventional microfiber sensors. The compact size, low cost, and immunity to electromagnetic interference make GMHW ideal for gas detection applications. Furthermore, a compact NH_3_ sensor featuring platinum-decoated graphene oxide (Pt/GO) improves sensing performance [63]. The addition of Pt nanoparticles facilitates a stronger attachment of NH_3_ to the GO matrix, resulting in a more pronounced change in the refractive index and enhanced reaction signals. This sensor exhibits a sensitivity of 10.2 pm/ppm, which exceeds the sensitivity of pure GO sensors by over three times, while maintaining linear measurements up to 60 ppm with effective recovery. The peak sensitivity was achieved with a Pt concentration of 185.2 mg/L, indicating optimal performance under these conditions. These findings demonstrate that Pt/GO nanocomposites can effectively detect NH_3_ gas at room temperature and safely in passive light conditions. Additionally, this study introduced a new NH_3_ gas sensor made from fluorinated graphene that turns dark yellow when it detects NH_3_ in the air [64]. It achieves high sensitivity at 4.05% ppm^−1^ of PL at 102 ppm of NH_3_. DFT calculations demonstrated that the presence of F atoms in graphene enhances both adsorption and charge transfer, enabling rapid detection within 20 min, with sensitivity and selectivity at 1 ppm concentration. Unlike other sensors, this design does not require any fabrication or electricity, highlighting its potential for cost-effective NH_3_ detection. Interestingly, a novel study demonstrated that the FLMG-100 sensor detects NO_2_ with exceptional sensitivity and effectiveness at room temperature [65]. This gas detection process of the FLMG-100 sensor shows a resistance change of nearly 16% when the NO_2_ gas concentration reaches 0.5 ppm, with the capability to detect NO_2_ concentrations as low as 25 ppb. High humidity levels (33% RH) and minimal reaction to NH_3_ (1.5%) indicate that the FLMG-100 sensor’s stability and selectivity are linked to its hydrophobic properties and the numerous gaps in its carbon structures. Therefore, the development of the FLMG-100 sensor positions it as a strong candidate for environmental and health monitoring systems. On the other hand, the SnS_2_/graphene composite sensor demonstrates superior performance compared to each individual material at room temperature when sensing NH_3_ [66]. At a concentration of 500 ppm NH_3_ in the air, its response reaches 91.32%, significantly surpassing that of the thin films alone. The optimized SnS_2_/G-b [(0.4 wt%) 1 mg graphene] fusion junction shows exceptional sensitivity, capable of detecting extremely low quantities as little as 0.1 ppm, operates swiftly, and sustains its performance over a period of 15 days with only minimal degradation. Additionally, density functional theory analyses suggest that SnS_2_’s ability to absorb larger amounts of NH_3_ gas, coupled with the rapid carrier mobility and efficient charge transfer of graphene, are key factors in its effectiveness. Moreover, the detection of NH_3_ is significantly improved using PANI-rGO nanocomposite membranes at room temperature [67]. A 125:1 PANI to rGO ratio yielded a maximum responsivity of 13% for 15 ppm NH_3_, demonstrating impressive response (1.6 min) and recovery (22.1 min) times. These benefits exceed those of standalone PANI or rGO membranes due to their synergistic effects, which result in increased surface area, uniformly distributed PANI, and optimized electron transport. Furthermore, the PANI-rGO nanocomposite membrane sensors can detect gas concentrations as low as 0.3 ppm and maintain stability across a wide range of humidity levels, elucidating their potential for effective environmental and health monitoring. Captivatingly, the GO/TiO_2_ nanocomposites demonstrated markedly superior performance in NH_3_ sensing at room temperature [68]. The GT-5 sensor achieved a 96% response to NH_3_ at 100 ppm, displaying quicker reaction (13 s) and recovery (33 s) times than pure GO. The integration of the GO/TiO_2_ junction, along with increased surface roughness and embedded TiO_2_, creates more active sites and enhances electron mobility within the graphene oxide/titanium dioxide hybrid network. Density functional theory (DFT) verified that GO/TiO_2_ provides superior NH_3_ adsorption and enhances electron transfer at the junction, rendering it effective for NH_3_ detection and identification.

Innovative techniques for ammonia gas detection have emerged by utilizing advanced and hybrid materials, tackling challenges related to sensitivity, selectivity, and functionality of ammonia at room temperature. Interestingly, a composite of Cu(INA)_2_ and reduced graphene oxide (rGO) on interdigital electrodes effectively detects NH_3_ gas [69]. The sensor consistently delivers results as concentration levels increase from 50 ppm to 500 ppm. The Cu(INA)_2_ metal–organic framework (MOF) exhibits a porous structure, and its strong affinity for NH_3_ is attributed to the coordination of NH_3_ at the Cu^2+^ center. Additionally, rGO provides a conductive surface that enhances the mobility of charge carriers. The adsorption of NH_3_ catalyzes a synergistic effect between these two elements, enhancing electron transfer efficiency and resulting in notable changes in resistance. The robust interplay between the creation and reduction in sensing molecules during operation ensures structural resilience and exceptional performance, thereby paving the way for enhanced NH_3_ sensors. In a different study, a hybrid sensor utilizing polyaniline (PANI) and Fe-doped cerium oxide (CeO_2_) was developed to enhance sensitivity at room temperature [70]. This enhancement occurs in two steps: ammonia molecules function as bases that extract protons from PANI, leading to reduced charge carrier mobility and increased resistivity. At the same time, incorporating Fe into CeO_2_ generates oxygen vacancies and creates active sites for electron transfer, facilitating NH_3_ adsorption and improving NH_3_-N_2_ conversion through Ce^4+^/Ce^3+^ and Fe^3+^/Fe^2+^ redox cycles. Therefore, the combination of conductive polymers with doped metal oxides presents a promising strategy for achieving sensitivity at room temperature. In contrast, integrating boron modifies the physical and chemical structures of graphene, resulting in B-doped graphene nanosheets [71]. The electrons associated with boron attract electron-rich NH_3_ molecules, which impart p-type conductivity to the graphene lattice. NH_3_ interacts with the boron-doped few-layer graphene (BFLGr) surface by donating electrons, which raises resistance by reducing the number of hole carriers. As a result, the BFLGr sensor is fast, easily reversible, and efficiently detects specific gases in ordinary environments, illustrating that the modification of graphene with heteroatoms is a viable and effective approach for gas detection. Fascinatingly, a novel hybrid field-effect transistor (FET) was fabricated using TiO_2_/graphene to improve dynamic sensing [72]. This approach enabled the TiO_2_/graphene devices to switch between different types of semiconductors, allowing for adjustable responses to donor gas, such as NH_3_. The sensor showed a response of 16.4% to 25 ppm NH_3_, with complete recovery after exposure to moderate humidity, as its entire surface was coated with TiO_2_. The low work function of the gate, combined with the electron-rich characteristics of TiO_2_, shifted the Fermi level, facilitating effective electron exchange and strong interaction with NH_3_ gas. Additionally, increased humidity levels facilitated the adsorption and desorption process. Therefore, the gas sensing process offers both flexibility and accurate sensing, making it suitable for a low-cost NH_3_ gas sensor. Overall, these studies suggest that modifying the electronic properties of gas sensing materials can enhance their interaction with NH_3_ molecules at the surface at room temperature. It is concluded that each strategy, such as synergistic, doped, or gate-controlled sensors, offers unique improvements in NH_3_ gas sensing performance. Furthermore, ammonia gas sensing with nanolayer-decorated epitaxial graphene (DEG) shows improved sensitivity and selectivity through surface modifications [73]. In this research, DEG sensors coated with metal oxide nanolayers like CuO, Fe_3_O_4_, V_2_O_5_, and ZrO_2_ were evaluated for NH_3_ detection in different environmental conditions. The sensors behaved differently depending on the metal oxide used, temperature, and humidity levels. Notably, ZrO_2_-based sensors showed the highest NH_3_ response at 125 °C (21.2% for 25 ppm), while others reached peak performance at lower temperatures, around 50–75 °C. Generally, increased humidity decreased the sensor response, except for V_2_O_5_, which became more sensitive. UV light, especially at 355 nm, improved responses and shortened response times for most sensors. These findings suggest that integrating epitaxial graphene with metal oxides and adjusting environmental factors can effectively enhance NH_3_ sensing, supporting reliable air quality monitoring and similar applications.

**Figure 3:**
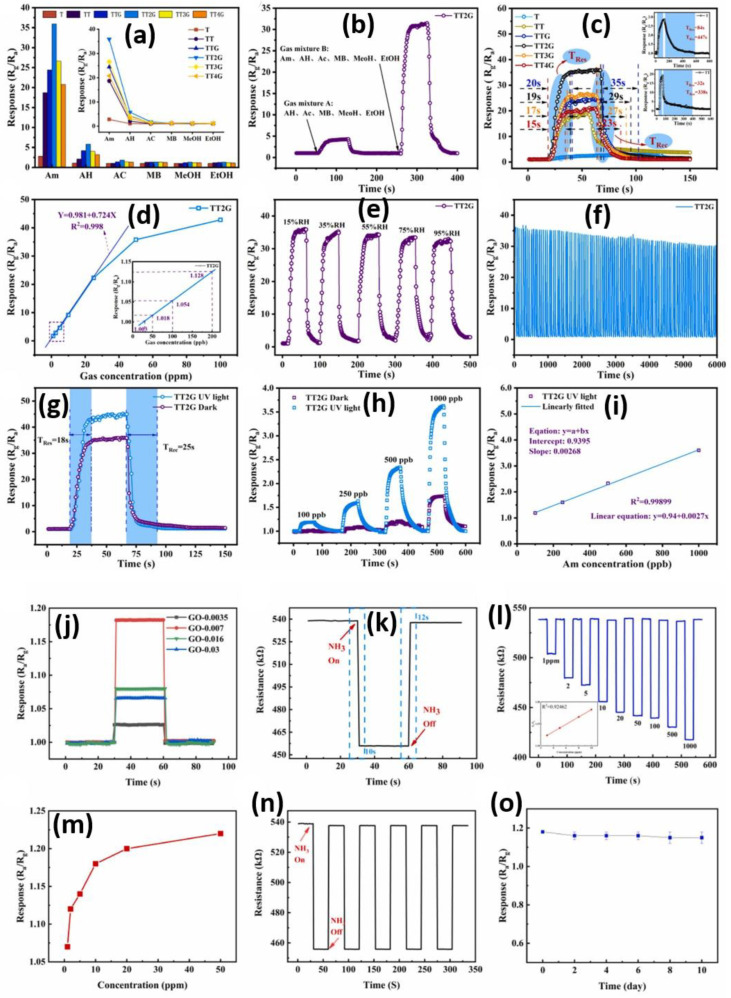
Comprehensive evaluation of the NH_3_ gas sensing performance of Ti_3_C_2_T_x_/TiO_2_/graphene (TT2G) sensors. (**a**) Selectivity of various sensors to 50 ppm of different gases at 35% RH and 25 °C under dark conditions, showing TT2G’s strong preference for NH_3_. (**b**) Response of the TT2G sensor to a gas mixture with and without 50 ppm NH_3_. (**c**) Dynamic response and recovery behavior of different composites at 50 ppm NH_3_. (**d**) Sensitivity of TT2G to NH_3_ concentrations ranging from 1 to 100 ppm, with good linearity at low concentrations. (**e**) Influence of relative humidity (15–95% RH) on the response to 50 ppm NH_3_, demonstrating excellent humidity tolerance. (**f**) Repeatability and stability assessment of the TT2G sensor over 100 sensing cycles at 50 ppm NH_3_. (**g**) Effect of UV illumination on response/recovery behavior for 50 ppm NH_3_, showing enhanced performance. (**h**) Real-time response of the TT2G sensor to sub-ppm NH_3_ levels (100–1000 ppb) under UV light. (**i**) Linear fit of sensor response versus NH_3_ concentration under UV illumination, indicating a detection limit of 22.23 ppb. Reproduced with permission from Ref. [74]. Copyright (2024) Elsevier. (**j**–**o**) Gas sensing performance of the SiO_2_/GO/PVA hydrogel sensor toward NH_3_ at 25 °C. (**j**) Response to 10 ppm NH_3_ as a function of GO content, showing optimal performance at 0.007 mg/mL GO concentration. (**k**) Transient response and recovery behavior of the sensor toward 10 ppm NH_3_, exhibiting rapid kinetics with response and recovery times of 10 s and 12 s, respectively. (**l**) Dynamic response curves of the sensor to NH_3_ concentrations ranging from 1 to 1000 ppm. (**m**) Relationship between sensor response and NH_3_ concentration, illustrating saturation behavior at higher concentrations. (**n**) Repeatability test over five exposure cycles to 10 ppm NH_3_, confirming high reproducibility. (**o**) Long-term stability assessment of the sensor response to 10 ppm NH_3_ over 15 days, demonstrating excellent durability and consistency. Reproduced with permission from Ref. [75]. Copyright (2025) Elsevier.

Combining graphene with metal oxides and other nanomaterials stands out as a highly effective method for improving gas sensing capabilities. The features of increased surface area, enhanced charge transfer, and additional active sites often lead hybrid, composite, or heterostructure gas sensing materials to achieve superior sensitivity, enhanced selectivity, and greater responsiveness. A prominent recent trend is the utilization of ternary nanocomposites, which synergistically conglomerate the unique properties of each material. Figure 3a–i illustrates that the Ti_3_C_2_T_x_/TiO_2_/graphene (TT2G) sensor detects ammonia significantly more effectively compared to other sensors [74]. The TT2G sensor demonstrates impressive functional capabilities in selectivity, response, detection limits, temperature resilience, and humidity tolerance, making it suitable for breath analysis. In Figure 3a, the TT2G sensor exhibits a robust reaction to NH_3_ compared to other chemicals like acetaldehyde, toluene, acetone, methanol, and ethanol, as indicated by its considerably higher response values. Figure 3b shows that the sensor predominantly identifies NH_3_, even amidst mixed gases, highlighting its capability to handle the intricacies of breath analyses. The integration of graphene with Ti_3_C_2_T_x_/TiO_2_ boosts charge transfer between the sensor layers, leading to a greater number of active adsorption sites that enhance accuracy and minimize response times. Among all sensors, TT2G demonstrates the highest response (35.8) and the quickest rise and fall times (19 s and 29 s) for 50 ppm NH_3_, outperforming the pristine Ti_3_C_2_T_x_ and Ti_3_C_2_T_x_/TiO_2_ sensors, as shown in Figure 3c. The ternary structure and the arrangement of the Ti_3_C_2_T_x_/TiO_2_/graphene (TT2G) sensor enhance both electron transport and NH_3_ absorption. Notably, Figure 3d,i show that the TT2G sensor exhibits a near-linear response across a broad range of NH_3_ concentrations (from 1 ppm to 1000 ppb), making it a reliable choice for quantification. Without UV assistance, the method can detect as little as 26 ppb of NH_3_ in breath analyses; however, the sensor detection limit improves to 22.23 ppb with UV assistance. In Figure 3e, the TT2G sensor demonstrates effective operation at 95% relative humidity. It exhibits consistent accuracy even after every hundred cycles [Figure 3f], allowing for continuous monitoring. Furthermore, the use of UV in Figure 3g–i significantly boosts both the response and response time (1.54 to 45.2 S for 50 ppm NH_3_), indicating that UV-assisted photoactivation substantially facilitates the interaction of gas molecules and enhances the dynamics of surface charges. Together, the TT2G sensor marks a significant advancement in the detection of ammonia gas at ambient temperatures, owing to its rapid reactions, high sensitivity, strong selectivity, and adaptability in various conditions. Similarly, Figure 3j–o presents the gas sensing behavior of another ternary sensing SiO_2_/GO/PVA hydrogel material toward NH_3_ at 25 °C [75]. Figure 3j shows that the hydrogel’s response to NH_3_ varies based on the amount of graphene oxide (GO) present. The sensor’s reaction to GO increases, peaking at 0.007 mg/mL, before declining as GO concentration rises. At high GO concentrations, some aggregates form, resulting in reduced active adsorption space and fewer NH_3_ molecules being adsorbed. Figure 3k illustrates the response and recovery time at 10 ppm NH_3_. The rapid adsorption and desorption are primarily due to the porous structure of SiO_2_ combined with the surface functional groups on GO. Figure 3l illustrates the sensor’s dynamic response across a broad spectrum of NH_3_ concentrations (1–1000 ppm). As the NH_3_ concentration increases, the response intensifies, indicating a strong correlation with the amount. Notably, the sensor can detect contamination levels as low as 1 ppm, which is critical for real-world applications in agriculture, industry, and environmental monitoring. Figure 3m depicts a saturated response, which occurs when the NH_3_ concentration exceeds the hydrogel’s capacity, resulting in a decrease in the active oxygen species on its surface. The linearity within the 3–10 ppm range is perfect for use in calibration and quantitative analysis. The repeatability of the sensor at a concentration of 10 ppm NH_3_ was assessed over a period of 5 cycles, as illustrated in Figure 3n. The stable values and well-matched responses from baseline to peak confirm that the sensing mechanism stays the same and reversible, showing almost no degradation despite repeated use. When exposed to 10 ppm NH_3_ for 15 days, the sensor did not vary much in its response, indicating high stability, as presented in Figure 3o. These results suggest that SiO_2_/GO/PVA hydrogel-based sensors withstand changes in the surrounding environment and retain their core features when stored or used for a long period.

### 4.2. NO_2_ Gas Sensing

Nitrogen dioxide (NO_2_) arises from vehicle emissions, industrial combustion, and the burning of wood, and several other ways. Although present in small amounts, NO_2_ poses a significant threat to human health, contributing to respiratory ailments and cardiovascular diseases [76]. Its strong electron-accepting ability allows NO_2_ to interact easily with graphene and other p-type semiconductors, causing notable variations in conductivity [77]. The combination of graphene’s extensive surface area, high electron mobility, and thin atomic structure makes it exceptionally responsive to NO_2_. Nevertheless, standard graphene exhibits limited selectivity and poor recovery, prompting researchers to explore doped graphene, metal oxides, and 2D hybrids to enhance its performance under typical conditions. Consequently, numerous researchers are now creating hybrid materials that integrate graphene with other materials to improve sensitivity, selectivity, and recovery. A notable concept is merging graphene with molybdenum disulfide (MoS_2_), a two-dimensional transition metal dichalcogenide known for its semiconducting properties. The combination of MoS_2_ and CVD-grown graphene showcased excellent NO_2_ sensing performance at room temperature, with the ability to detect concentrations lower than 50 ppb [78]. This impressive capability resulted from improved electron transport within the hybrid and the exposure of molybdenum edges through thermal annealing. The smooth graphene substrate promoted even nanoparticle distribution and enhanced surface accessibility, which facilitated effective NO_2_ adsorption and desorption. The hybrid sensor showed greater selectivity and recovery in humid conditions compared to MoS_2_ on other substrates, with its advantages stemming from the interplay between graphene’s conductivity and the active edge sites of MoS_2_. On the other hand, by combining zirconium and graphene oxide, a ZIF-67/rGO nanocomposite improved the detection of NO_2_ gas at room temperature [79]. The ZIF-67/rGO sensor produced a response that was 13 times greater than that of pure rGO, while its detection limit was only 0.5 ppm. The benefits of MOF’s large surface area and numerous pores, along with rGO, enhanced its conductivity and enabled rapid charge transport. Due to the hybrid structure, the ZIF-67/rGO sensor reacted quickly (15 s) and returned to its initial state in 40 s, demonstrating good selectivity and consistent performance each time. Benefits from MOF’s great surface area and many pores, and rGO contributed to enhancing the conductivity and fast charge transport in ZIF-67/rGO sensor. Interestingly, the rGO@In_2_O_3_ nanofiber-based flexible gas sensors, fabricated through coaxial electrospinning and thermal treatment, demonstrated excellent NO_2_ detection at 25 °C [80]. The rGO@In_2_O_3_ nanofiber-based flexible gas sensor exhibited high selectivity and durability, achieving a measurement of 14.18 at 1 ppm NO_2_ and detecting as low as 2.58 ppb of NO_2_. The formation of a p-n heterojunction and a depleted surface layer at the rGO and In_2_O_3_ interface led to an accumulation of more charge carriers, enhancing surface reactivity. With their adaptability and dependable performance, these wearable and portable rGO@In_2_O_3_ nanofiber-based flexible gas sensors can explore new possibilities for industrial and public safety. Furthermore, a novel graphene-TiO_2_ structure ornamented with Au sensors was degined, which can specifically detect NO_2_ through simple light activation [81]. Under UV light (275 nm), the Au@graphene-TiO_2_ sensor achieved a detection limit of 10 ppb, with increased sensitivity observed under green light (515 nm) due to localized surface plasmon resonance (LSPR) at the Au sites. The response spectra demonstrated that light exposure influences charge carriers, resulting in an increase in the width of their Lorentzian features based on NO_2_ concentration. Moreover, a novel gas sensing material crafted from V-MOFs on flexible graphene and infused with terephthalic acid displayed exceptional sensitivity and selectivity to NO_2_ [82]. V-MOF_120_(PTA) sensor demonstrated an excellent response of 800.8% for 100 ppm NO_2_, reacting within 230 s and recovering in 39.8 s. It successfully detected as low as 1 ppm NO_2_ while ensuring reliable performance. The approach employed in this study yielded materials with high surface area and porosity, thereby promoting gas adsorption and electron flow. It is concluded that attaching MOFs to flexible substrates can yield high-performance and low-cost NO_2_ sensors suitable for wearable technology. Furthermore, a first-principles study demonstrated that doping graphene with gold (Au) and boron (B) enhances the selective detection and sensing performance of NO_2_ gas [83]. Graphene doped with an alkaline earth metal showed a strong affinity for NO_2_, resulting in a shift in its electronic properties, which indicates its potential for use in devices reliant on resistance or work function. In contrast, B-doped graphene mainly displayed physisorption during NO_2_ gas detection. These findings highlight that modifying the doping levels in graphene can influence NO_2_ adsorption and alter the bandgap, both of which are essential for the detection of NO_2_ and similar gas sensing applications. Interestingly, a low-cost NO_2_ gas sensor was developed by applying reduced graphene oxide on commercial cotton fabric [84]. The reduced graphene oxide@cotton fabric showed the best sensing performance (45.9% at 10 ppm NO_2_) at 190 °C, which is primarily attributed to its high number of oxygen functionalities and porous structures. The sensor’s response diminished at lower temperatures, resulting in a decrease in the density of functional groups. This e-textile design could facilitate the mass production of wearable graphene sensors that function well at standard room temperatures, due to its flexibility, ease of production, and adequate sensitivity. Stimulatingly, gas sensors made from a combination of p-type reduced graphene oxide and n-type Gallium oxide nanorods exhibited a 51.14 response to nitrogen dioxide (NO_2_) [85]. The p-n heterojunction facilitated efficient charge transfer, resulting in increased oxygen vacancies that improved the sensing material’s reactivity and charge mobility. This approach demonstrates the potential to create sensing devices by integrating metal oxide materials with carbon materials, thereby enhancing both sensitivity and operational efficiency. Attractively, a novel computational study revealed that boron and arsenic-doped armchair graphene nanoribbon (ArGNR) sensors have a robust reaction with NO_2_, leading to the formation of N_2_, which induces stable changes in their bandgap of up to 100% and current of up to 95% [86]. Incorporating B atoms into silver graphene nanoribbons significantly enhances performance, reaching a 57% quantum transport efficiency. These studies underscore the crucial role of these two aspects in determining sensor effectiveness. On the other hand, a surface acoustic wave (SAW) sensor featuring palladium-modified reduced graphene oxide (Pd-rGO) film exhibited exceptional NO_2_ sensing capabilities at room temperature, achieving a sensitivity of 146.9 Hz/ppm and a detection limit of about 1 ppm [87]. The spin-coated Pd-rGO layer provided a broad surface area and numerous active sites, while palladium enabled the catalytic breakdown of NO_2_. The sensor exhibited remarkable selectivity toward unwanted gases and maintained consistent performance over extended periods. This study highlights the combined advantages of palladium and rGO’s conductivity in SAW-based sensors, illustrating their effectiveness for real-time environmental monitoring.

Furthermore, a new nanocomposite, Fe_3_O_4_@rGO-N-(piperidine-4-SO_3_H) sensor, has been created for NO_2_ gas sensing applications [88]. The Fe_3_O_4_@rGO-N-(piperidine-4-SO_3_H) sensor was developed in four steps and tested across various concentrations of NO_2_, ranging from 2.5 to 50 ppm. It exhibited optimal sensitivity for NO_2_ concentrations (≥5 ppm) with 15 wt% rGO concentration Fe_3_O_4_@rGO-N-(piperidine-4-SO_3_H). Due to the swift and enhanced interaction with NO_2_ molecules, the Fe_3_O_4_@rGO-N-(piperidine-4-SO_3_H) sensor was able to respond quickly to changes, thus improving its performance. Therefore, it is concluded that the study highlights the significant role that multifunctional graphene can play in detecting low-level NO_2_. On the other hand, incorporating graphene into SnO_2_ nanoparticles enhanced the detection of NO_2_ at ordinary room temperatures [89]. Ultraviolet light facilitated charge movement, enabling the device to identify trace amounts of NO_2_ amidst other gases. The introduction of graphene significantly boosted the SnO_2_/graphene sensor’s capacity to recognize and select NO_2_ while reducing its sensitivity to other gases. In addition, it revealed a 600% increase in response when the sensor was exposed to 0.5 ppm NO_2_. This illustrates that both photoactivation and material combination are crucial for swift and energy-efficient pollutant detection, which is essential for monitoring air quality in urban cities. Interestingly, a novel Bl-Gr-LiTaO_3_-S-Gr trio was developed on a surface acoustic wave (SAW) device for NO_2_ sensing [90]. S-Gr achieves a sensitivity limit of 1.17°/ppm and a detection limit of 0.136 ppm, surpassing Bl-Gr’s 0.29°/ppm and 0.068 ppm. Both sensor types functioned well across different humidity levels and gas environments, although Bl-Gr showed superior selectivity. Therefore, it suggests that the Bl-Gr-based SAW sensors are efficient for monitoring environmental NO_2_ at room temperature, small, quiet, and power-free. A wearable monitoring device was developed incorporating NFC technology, graphene sensors, and ZnO nanoflowers [91]. This sensor is designed to operate continuously for an entire day with minimal power consumption, continuously monitoring NO_2_ levels. Among the tested sensors, the ZnO on Kapton graphene-based sensor exhibited the best sensitivity and selectivity across all conditions, including both dry and humid environments. This advancement offers safety and cost benefits by integrating flexible electronics that monitor NO_2_ gas in real time. Fascinatingly, the integration of TiO_2_ via laser evaporation with graphene led to the creation of a highly sensitive and reliable system for detecting NO_2_ at room temperature [92]. By employing 365 nm UV light, the sensor quickly identified low NO_2_ concentrations. It revealed that theoretical modeling demonstrated the junction’s ability to promote effective charge transfer. Unlike visible light, UV radiation significantly improved the adsorption and desorption processes. The integrated TiO_2_/graphene sensor exhibited minimal cross-reactivity with CO, NH_3_, and SO_2_, as well as a stable nature, confirming its effectiveness for detecting low levels of NO_2_ in smart sensor applications. In the other case, graphene nanosheets extracted from recycled PET plastic were utilized to detect NO_2_ at room temperature [93]. The GO sensor exhibited full reversibility and could detect NO_2_ concentrations as low as approximately 1.43 ppb, with a response of about 11% at 1026 ppb of NO_2_. In contrast to NO_2_, the sensor showed much lower sensitivity to CH_4_, H_2_, and CO_2_. The sensing mechanism employed physisorption and charge-transfer-induced dipoles, eliminating the need for external excitation. The mixture remained stable and consistent for five consecutive days, highlighting the potential for developing high-performance NO_2_ sensors with low energy requirements. Additionally, the detection of NO_2_ gas was studied by utilizing MnO_2_/GO composites, which respond sensitively to different humidity levels [94]. The MnO_2_/GO composite sensor showed a response change of 16.3% in dry conditions and 44% at 70% relative humidity (RH) for 1 ppm NO_2_ at 150 °C. The synergy between MnO_2_ and GO enhanced electron transfer, leading to more effective interactions with NO_2_. It exhibited excellent selectivity against gases, such as NH_3_, CO, ethanol, and H_2_. Therefore, these findings suggest that the combination of MnO_2_ and GO produces reliable NO_2_ sensors that are well-suited for standard outdoor conditions. Interestingly, microporous polymers (PIM-1, PIM-EA-TB, and Matrimid) improved the detection capabilities of graphene pixel array sensors for NO_2_ [95]. The application of PIM coating enhanced sensitivity by −25.7%, whereas bare graphene achieved −10.8%, and the response time was quicker by 56 s. The PIM-EA-TB detected NO_2_ successfully at 0.7 ppb. These polymers excelled in gas molecule capture and showed minimal influence from NH_3_, CH_4_, and CO_2_. Consequently, polymer-modified graphene sensors are capable of real-time NO_2_ monitoring with low power consumption, underscoring the significance of PIMs in the advancement of graphene sensors. Instead, the detection of NO_2_ using N/B-doped rGO sensors improved at 100 °C, with N-rGO showing the most incredible sensitivity and the ability to detect at the lowest concentration (below 1 ppm) of NO_2_ [96]. Chemical doping adjusted the sp^2^ networks and created new bonding opportunities (B-C_3_ and pyridinic-N), which positively influenced desorption and charge transfer. Undoped GO could not achieve the performance compared with N/B-doped rGO sensors, thereby allowing for the development of NO_2_ sensors that are compatible with CMOS, have low energy consumption, offer improved selectivity, and work effectively in different conditions. Soydan et al. developed room temperature NO_2_ sensors by utilizing laser-induced graphene (LIG) combined with SnO_2_ heterostructures [97]. This single-step laser process created conductive LIG within the pores of polyimide films, significantly enhancing gas adsorption. The sensitivity and selectivity of the LIG/SnO_2_ mixture toward NO_2_ benefited from the synergistic charge transport within its structure and porosity. Importantly, these sensors can monitor air quality without needing external heating, providing a technique that is both power-efficient and cost-effective. This suggests that LIG sensors are highly suitable for environmental monitoring applications. On the other hand, Epitaxial graphene (EG) grown on SiC substrates shows exceptional gas sensing capabilities, especially for NO_2_ [98]. Its high sensitivity results from strong interactions between NO_2_ molecules and the n-type doped graphene surface, causing notable resistance changes. Defect engineering techniques, like SF_6_ plasma treatment, further improve sensitivity by increasing adsorption sites. Among the tested samples, EG achieved a maximum response of 105.1% at 4 ppm NO_2_, with a detection limit of just 1 ppb. This performance exceeds that of quasi-free-standing and CVD epitaxial graphene, underlining EG’s potential for creating ultra-sensitive, low-detection-limit gas sensors. Interestingly, epitaxial graphene (EG) shows outstanding gas sensing abilities because of its large surface area and minimal electronic noise [99]. When grown on SiC substrates, EG usually behaves as an n-type material due to charge transfer from the buffer layer. Exposure to NO_2_ causes a notable change in resistance, allowing detection at levels below 1 part per billion. Graphene responds quickly and strongly to NO_2_ because of its high charge transfer sensitivity. Furthermore, coating it with NiO leads to a switch to p-type conduction by modulating the Fermi level across the Dirac point. This hybrid NiO/graphene system provides highly sensitive, reversible NO_2_ detection with excellent selectivity and reproducibility.

**Figure 4:**
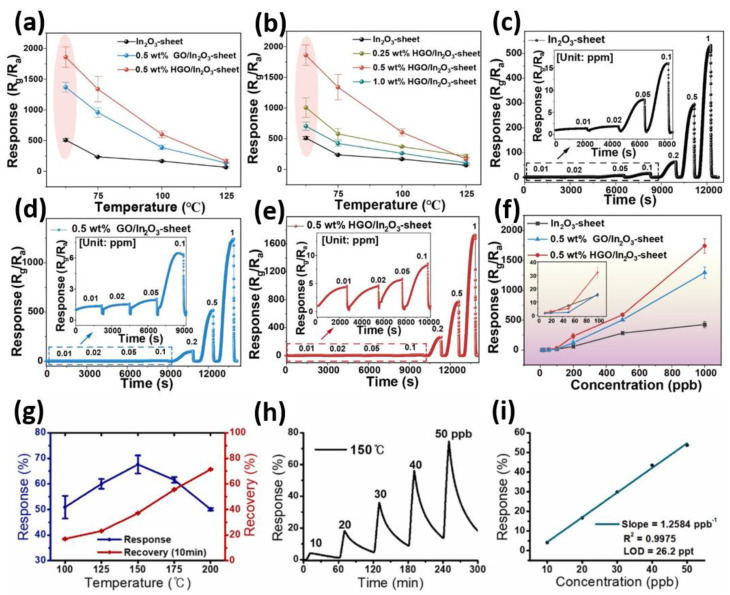
Gas-sensing performance of holey graphene oxide (HGO)/In_2_O_3_-based sensors. (**a**) Responses to 1 ppm NO_2_ for In_2_O_3_-sheet, GO/In_2_O_3_-sheet, and HGO/In_2_O_3_-sheet sensors, depending on temperature, (**b**) Effect of HGO loading on sensor response at 62.5 °C, (**c**–**e**) Dynamic response and recovery curves for various NO_2_ concentrations, (**f**) Response compared to NO_2_ concentration, demonstrating detection ability down to 10 ppb. Reproduced with permission from Ref. [100]. Copyright (2024) American Chemical Society. (**g**) Temperature-dependent response and recovery behavior of the rGO/SnO_2_ sensor to 100 ppb of NO_2_ under dry conditions, (**h**) dynamic response curves of the rGO/SnO_2_ sensor to varying NO_2_ concentrations (10–50 ppb) at 150 °C, (**i**) corresponding calibration curve showing a linear sensor response and a calculated LOD. Reproduced with permission from Ref. [101]. Copyright (2025) Elsevier.

Advancements in hybrid nanomaterials have significantly improved the detection performance for NO_2_. Graphene-based materials, particularly HGO/In_2_O_3_ and rGO/SnO_2_, have become noteworthy for their high surface area, electrical modifiability, and excellent adsorption characteristics. Extensive research has been conducted to elucidate their performance at various temperatures and concentrations. This analysis examines the efficiency of HGO/In_2_O_3_ and rGO/SnO_2_ as gas sensors, providing in-depth insights into their response, sensitivity, reversibility, and detection thresholds, as discussed in Figure 4a–i. Interestingly, Figure 4a–f depicts the temperature-sensitive gas-sensing properties of HGO/In_2_O_3_ composites [100]. In Figure 4a, the HGO/In_2_O_3_-sheet shows a response of 1859 at 62.5 °C and 1 ppm NO_2_, surpassing the response of the pure In_2_O_3_-sheet and GO/In_2_O_3_ by factors of 3.64 and 1.37, respectively. This demonstrates a significant enhancement in NO_2_ sensing due to HGO modification. Figure 4b reveals that the ideal amount of HGO is 0.5 wt%, as deviations in quantity lead to diminished sensitivity caused by increased surface recombination or lowered electrical conductivity. The sensors exhibit rising resistance with increasing NO_2_ concentration, as illustrated in Figure 4c–e, confirming their classification as n-type semiconductors. Notably, the 0.5 wt% HGO/In_2_O_3_ sensor responds swiftly and consistently returns to its original state, showcasing exceptional sensitivity and reversibility. Figure 4f displays the calibration curve for NO_2_ concentration from 10 ppb to 1 ppm, showing that the 0.5 wt% HGO/In_2_O_3_ sensor responds in a very steep and consistent way. These results indicate that adding HGOs increases the number of active adsorption sites and enhances electrical transfer, resulting in improved NO_2_ detection in ambient air. Conversely, the rGO/SnO_2_ sensor exhibits enhanced NO_2_ sensitivity, influenced by temperature and concentration as illustrated in Figure 4g–i [101]. Figure 4g represents the response and recovery times for 100 ppb NO_2_ at temperatures ranging from 100 to 200 °C. The findings reveal that the response at 150 °C achieves 67.6% while leaving a recovery of 37.1%. This optimal temperature likely facilitates favorable reactions between NO_2_ molecules and the composite, enhancing the speed of adsorption without triggering premature desorption. As temperatures rise from 150 to 400 °C, the response diminishes, indicating that swift gas desorption at such elevated temperatures may shorten the time available for interactions with the gas sensor. Figure 4h shows the NO_2_ concentration curves over time for 10, 20, 30, 40, and 50 ppb. The rGO/SnO_2_ exhibits a linear response to increasing n-NH_2_ levels, even starting at 10 ppb, which is significantly greater than the response of unmodified rGO. This linearity is further supported by Figure 4i, which presents a well-fitted calibration curve with an exceptional limit of detection (LOD) of 26.2 ppt, indicating ultra-sensitive performance. The n-type SnO_2_ layer enhances NO_2_ adsorption, increasing the electrical resistance of the p-type rGO by withdrawing electrons. Interestingly, Figure 4g–i demonstrate that the treatment of rGO with SnO_2_ facilitates NO_2_ sensing by offering a strong response, rapid recovery, and good sensitivity, particularly with appropriate thermal treatment. These findings provide a crucial comparative insight that the HGO/In_2_O_3_ enables energy-efficient NO_2_ sensing with excellent responsiveness at ambient temperatures, whereas rGO/SnO_2_ demonstrates ultrasensitive detection under moderately elevated temperatures. This distinction demonstrates that modifying materials and operating conditions can effectively balance sensitivity, response, and detection limits.

### 4.3. VOCs Gas Sensing

Volatile Organic Compounds (VOCs) are chemicals that readily vaporize, impacting environmental and industrial safety, as well as various medical tests. Common VOCs, such as benzene, toluene, formaldehyde, ethanol, methanol, and acetone, are prevalent in the atmosphere and can cause health issues, such as lung irritation and cancer [102]. Therefore, prompt detection and quantification of VOCs are essential for air quality monitoring, maintaining safe workplaces, and facilitating early disease detection through breath tests [103]. Nonetheless, detecting VOCs in a selective and sensitive manner is still challenging because of their complex structures, low concentrations, and possible interference from other gases. This section reviews recent advancements, sensors, and performance improvements in VOC gas detection, with an emphasis on developments that enhance selectivity, sensitivity, rapid response, and long-term stability. The development of graphene-based hybrid sensors represents a significant breakthrough in detecting volatile organic compounds (VOCs), mainly due to their outstanding electrical, optical, and structural properties. Various studies indicate that the integration of graphene or its derivatives with other nanomaterials improves sensitivity, selectivity, and stability, particularly at room temperature, which is essential for real-world applications.

A graphene-ZnO sensor successfully identified ethanol at a low threshold of 100 ppb under standard room temperature conditions [104]. Zinc oxide served as an effective catalyst, while graphene enhanced electrical conductivity. The system demonstrated a response time of under 10 s, indicating high accuracy and reliable reproducibility. The combination of ZnO and graphene promoted improved charge transfer, creating a stronger signal for the sensor. This hybrid method enabled effective detection of ethanol vapor in ambient conditions, making it a promising solution for practical monitoring of volatile organic compounds. On the other hand, a ZnFe_2_O_4_-graphene nanocomposite sensor was designed for detecting volatile organic compounds [105]. This sensor outperformed traditional metal oxides in detecting ethanol and acetone at low temperature. Due to the graphene structure, electron movement was enhanced, which inhibited the collision and aggregation of nanoparticles. Its performance was about 8 to 10 times greater than that of ZnFe_2_O_4_ on its own, with capabilities to identify trace amounts at the ppm level. The ZnFe_2_O_4_-graphene nanocomposite sensor exhibited a rapid response and could be reused multiple times, demonstrating exceptional reliability. Therefore, graphene sensors significantly boost the effectiveness of VOC detection and withstand environmental variations. Interestingly, a sensor for ethanol detection has been developed by electrochemically coating nickel nanoparticles (Ni) onto Nafion/graphene films [106]. This sensor demonstrated remarkable activity, identifying a broad range of concentrations (0.43–88.15 mM) with a detection limit of 0.12 mM. Unlike conventional metal-based sensors, this sensor exhibited greater stability and can be utilized for recognition of ethanol molecules. This approach addresses the instability associated with enzymes, and the results suggest that Ni/graphene composites are promising candidates for ethanol sensing applications. Additionally, a SnO_2_-graphene sensor was created for formaldehyde detection at room temperature [107]. The incorporation of ZnO quantum dots into the graphene led to a significant performance boost for the SnO_2_-graphene sensor. It exhibits a rapid response time of 30–40 s, all while maintaining selectivity and stability. DRIFT spectroscopy indicated that when gas was added to ZnO, electrons transferred from ZnO to graphene, altering the mobility. This interaction between the layers negated the necessity for high temperatures in VOC sensing, thus making it suitable for indoor applications monitoring. Reduced graphene oxide (RGO) and RGO-rosebengal composites were utilized in resistive sensors for detecting ammonia, acetone, and ethanol [108]. It is indicated that RGO-RB’s response to ammonia was up to 36.6% higher compared to RGO alone. The sensor also exhibited notable sensitivity, capable of detecting ethanol in concentrations of 1.1–1.7% at room temperature. This study reinforces the notion that nanocarbon-based sensors can effectively select and accurately identify disease biomarkers during analyzing breath. Interestingly, a sensor has been developed utilizing ZnO quantum dots and graphene to measure formaldehyde at ambient temperatures [109]. Integrating 5 nm ZnO quantum dots improved both the reaction activity and charge transfer in graphene. This composite exhibited four times greater sensitivity compared to bare graphene. This sensor is highly effective for detecting air pollution, featuring a rapid response time of about 30 s and a recovery time of approximately 40 s, while also operating at room temperature and ensuring outstanding stability. Notably, a SnO_2_-reduced graphene oxide (RGO) composite sensor was developed for acetone detection through hydrothermal synthesis, demonstrating effective functionality at room temperature [110]. This SnO_2_-RGO composite sensor demonstrated a nearly double response compared to RGO, exhibiting enhanced response and recovery speeds, as well as improved repeatability. The combination of SnO_2_ and RGO provided the sensor surface with numerous active sites and efficient electrical conductivity. The high surface area and porous structure of the sensor significantly contributed to its effectiveness. The findings suggest that SnO_2_-RGO composites can be valuable for creating low-power VOC sensors. In addition, a sensor has been fabricated based on cataluminescence by combining SrCO_3_ and graphene to detect n-propanol [111]. The SrCO_3_/graphene sensor demonstrated a sensitivity 5.8 times greater than that of a bare SrCO_3_ sensor. It is capable of detecting a wide range of values (0.2–32 mg/L) with a lowest detection limit of 0.08 mg/L, providing instant response within just 2 s. The sensor proved highly accurate, showing favorable recovery rates for liquid samples spiked with n-propanol. It is concluded that the graphene and SrCO_3_ facilitated rapid cataluminescence on specific targets, indicating strong potential for VOC analysis. On the other hand, a sensor composed of reduced graphene oxide-polyethylene oxide (RGO-PEO) was created to identify toluene molecules [112]. The RGO-PEO sensor demonstrated greater sensitivity and faster response and recovery compared to both bare sensors. Performance remained unchanged with over 50% usage of PEO. At 50% PEO, the gas response for toluene was twice as high as that for ethanol, acetone, hydrogen, or formaldehyde. Due to its excellent selectivity and stability, the RGO-PEO platform is ideal for monitoring VOCs in ambient conditions. Moreover, a novel MLG sensor was fabricated to detect lung cancer biomarkers, such as acetone, ethanol, and isopropanol [113]. Distinctions between the biomarkers were evident at concentrations ranging from 1.4 to 2.0 × 10^5^ ppm, based on conductivity changes. The sensor’s suitability for non-invasive breath tests is highlighted by its effectiveness at room temperature, rapid detection capability, and reusability. However, several issues remain unresolved. Therefore, future research should concentrate on reducing size by integrating sensors with flexible materials and wireless technology suitable for portable or wearable devices.

**Figure 5:**
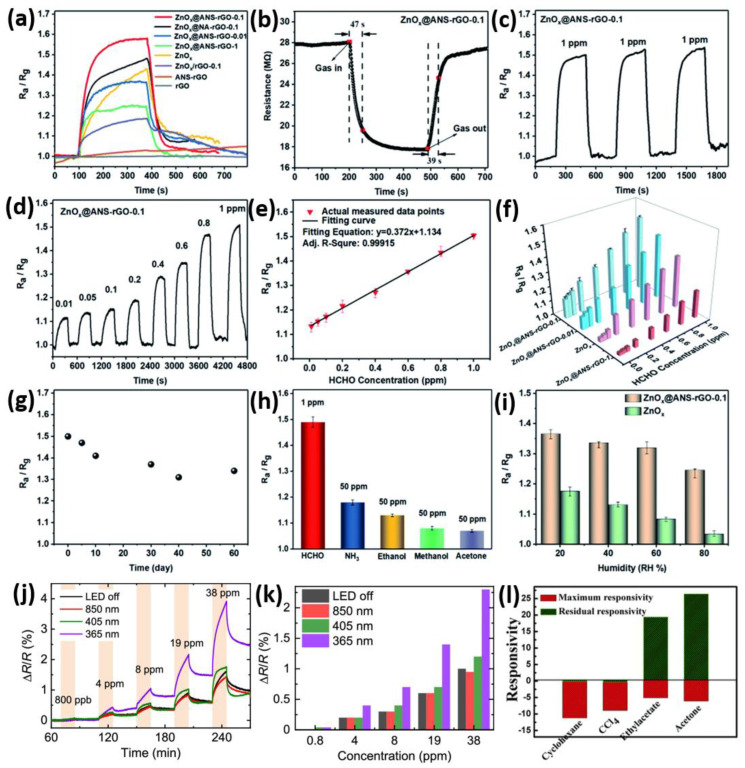
The detection of gases using ZnO_x_@ANS-rGO nanocomposites occurs under visible light at a 405 nm wavelength at room temperature. (**a**) The curves demonstrate the response of various sensing materials upon exposure to 1 ppm HCHO, (**b**) The ZnOx@ANS-rGO-0.1 illustrates the response and recovery times of HCHO, (**c**) The repeatable performance of the ZnO_x_@ANS-rGO-0.1 at 1 ppm HCHO, (**d**) Response plots of ZnO_x_@ANS-rGO-0.1 below 1 ppm HCHO, (**e**) A linear curve indicates the response of ZnO_x_@ANS-rGO-0.1 to varying concentrations of HCHO, (**f**) A comparison of measured values from different sensors with varying HCHO concentrations, (**g**) Stability for 1 ppm HCHO during 60 days, (**h**) Selectivity against various interfering gases at a fixed 50 ppm for all gases, and (**i**). Relative humidity levels ranging from 20% to 80% influence the sensor’s response for 0.5 ppm HCHO. Reproduced with permission from Ref. [114]. Copyright (2021) The Royal Society of Chemistry. GFET sensor response to varying concentrations of DMMP under different light wavelengths. (**j**) Real-time resistance changes under 365 nm (UV), 405 nm (near-UV), and 850 nm (IR) illumination, and (**k**) quantifies peak-to-peak relative resistance changes, highlighting enhanced sensitivity under UV due to photo-induced electronic modulation. Reproduced with permission from Ref. [115]. Copyright (2021) American Chemical Society. (**l**) Maximum and residual responsivities of TPU-graphene nanocomposites exposed to saturated organic vapors with varying polarities at 30 °C. Reproduced with permission from Ref. [116]. Copyright (2016) The Royal Society of Chemistry.

Figure 5a–i demonstrates that ZnOx@ANS-rGO-0.1 nanocomposites exhibit enhanced formaldehyde sensing capabilities at room temperature and under visible light at a 405 nm wavelength, attributed to the beneficial effects of oxygen vacancies and dipole modification [114]. The ZnO_x_@ANS-rGO-0.1 sensor exhibits the highest response for 1 ppm HCHO compared to the other sensors, as shown in Figure 5a. The connection between ZnO_x_ nanoparticles and ANS graphite sheets enables the formation of donor-π-acceptor dipoles, thereby enhancing electron conduction and providing an excellent sensor response. Figure 5b illustrates the response and recovery times of the ZnOx@ANS-rGO-0.1 sensor. Under 405 nm visible light at room temperature, the sensor exhibits rapid response and recovery times of 47 s and 39 s to 1 ppm HCHO, due to the surplus photogenerated charges. Additionally, the sensor successfully passes the repeatability test, as its output signal remains consistent across three exposure cycles, as displayed in Figure 5c. One significant advantage of this sensor is its ability to detect formaldehyde across various concentrations from 0.01 to 1 ppm, as illustrated in Figure 5d. Additionally, Figure 5e demonstrates an excellent correlation between the response and HCHO concentration, confirming its suitability for quantitative analysis and exhibiting 5 ppb low limit of detection (LOD). An analysis of different sensor configurations, as illustrated in Figure 5f, demonstrates optimal performance, due to the unique composition and properties of carbon nanotubes. Furthermore, a 60-day sensor’s stability evaluation, as depicted in Figure 5g, confirms the outstanding durability. Initially, during the first 10 days, the sensor experiences a decline in response due to moisture accumulation; however, thereafter, only a slight drift of 12% occurs, demonstrating the sensor’s long-term stability and reliability. As shown in Figure 5h, the ZnO_x_@ANS-rGO-0.1 sensor markedly improves its response to HCHO, detecting concentrations above 50 ppm while reducing response to other interfering gases. Furthermore, Figure 5i explores the effects of humidity, indicating a slight decline in sensor response as humidity increases from 20% to 80%. On the other hand, a novel graphene field-effect transistor (GFET) gas sensor is studied and exposed to various concentrations of DMMP under 365 nm (UV), 405 nm (near-UV), and 850 nm (IR) illumination, as shown in Figure 5j [115]. This indicates that significant sensing enhancement occurs not due to thermal effects or broadband optics, but rather from specific photo-induced mechanisms that function primarily at shorter wavelengths. Figure 5k quantifies this effect by illustrating the peak-to-peak relative resistance change for each illumination condition [115]. The sensor displays a ~50% increase in sensitivity at 365 nm and ~10% at 405 nm when compared to the dark condition, while no significant change occurs at 850 nm. These improvements are attributed to the UV-induced modulation of graphene’s electronic properties, due to the photodesorption of p-type impurities or charge transfer from defect states in the substrate. This optical doping alters the Fermi level, enhancing the GFET’s sensitivity near its Dirac point. Therefore, Figure 5j,k demonstrates that the UV illumination can effectively enhance sensitivity and facilitate wavelength-tunable selectivity in graphene-based gas sensors. Interestingly, Figure 5l illustrates the differences in maximum and residual responsivities of TPU-based composites (CPCs) containing 0.4 wt% graphene, evaluated using four saturated organic vapors [116]. In every instance, a negative vapor coefficient (NVC) is observed for all vapors, indicating that interface resistance decreases during the interaction of gaseous vapors with the sensors, which is the opposite of the typical positive vapor coefficient. The observed trend of cyclohexane (−11.2%) > CCl4 (−8.96%) > acetone (−6.06%) > ethyl acetate (−5.11%) confirms that weakly polar solvents yield the highest electron-transfer conduction. These findings demonstrate that nanocomposites exhibit high sensitivity, can differentiate based on polarity, and can toggle on and off in response to non-polar vapors, making them effective for repeated vapor sensing with polarity selectivity.

### 4.4. H_2_S Gas Sensing

Hydrogen sulfide (H_2_S) is a highly toxic and flammable gas that has a distinct sharp odor. Even at low concentrations, it can have detrimental effects on human health, particularly impacting the respiratory and nervous systems. Therefore, the development of sensitive and selective H_2_S sensors is essential, especially in industries such as petroleum refining, wastewater treatment, and biogas production. This review highlights advancements in enhancing material sensitivity to H_2_S, with a particular focus on sensors made from graphene and its composite materials.

Polyaniline/graphene nanocomposites doped with PSS demonstrated exceptional sensing capabilities for H_2_S, which is attributed to the high molecular weight of PSS that improved the conductivity and distribution of reduced graphene oxide [117]. The most favorable outcomes were observed in the 30 wt% graphene composite, featuring a minimum detection limit of 1 ppm and remarkable electrical conductivity of 168.4 S/cm. Enhanced π-π stacking and improved charge transport contributed to enhanced sensor responsiveness, enabling the material to be effectively utilized in flexible electrodes on PET surfaces. Furthermore, the detection of H_2_S at near-ambient temperatures was remarkably selective due to NiO nanodisks co-doped with boron and nitrogen on reduced graphene oxide [118]. The sensor exhibited a sensitivity range of 82 to 100 ppm H_2_S at 150 °C, achieving a low detection limit of 24 ppb. It outperformed bare NiO and undoped graphene mixtures due to the catalytically active sites formed by B and N-doping, coupled with improved conductivity, making it ideal for explosive environments and medical diagnostics. Additionally, the theoretical study employed DFT to analyze H_2_S adsorption on graphene, ZnO, BeO, and Ni-decorated graphene [119]. While pristine graphene showed weak physisorption, Ni-decorated graphene displayed robust chemisorption with an adsorption energy of −1.64 eV, attributable to the Ni-S covalent bonding. This indicates that Ni-decorated graphene has considerable potential for H_2_S detection applications, as the enhanced binding interactions and electron transfer processes establish favorable conditions for sensor development. Interestingly, ZnFe_2_O_4_ nanofiber-loaded electrospun RGO exhibited excellent H_2_S sensing capabilities, with the highest response being 147 to 1 ppm at 350 °C [120]. The best results were obtained with 1.0 wt% RGO and an annealing temperature of 600 °C. The sensors demonstrated selectivity to interfering gases such as NH_3_ and SO_2_. The enhanced response was attributed to the effects of the heterojunction, porous structure, and grain boundary, which facilitated gas adsorption and charge transfer. Furthermore, the significant H_2_S sensing results were observed in the 3D outcomes of reduced graphene oxide and hexagonal WO_3_ nanosheets created through hydrothermal methods [121]. The 3.8 wt% rGO/h-WO_3_ demonstrated a sensitivity of 168.58 at a concentration of 40 ppm of H_2_S, while the detection limit was recorded at 10 ppb at 330 °C. The porous structure provides efficient channels for gas diffusion, and the formation of heterojunctions with rGO enhances electron transfer and selectivity, making it optimal for detecting low concentrations of H_2_S. On the other hand, the combination of doping Ag nanoparticles with charged impurities on graphene sheets showed rapid response (4 s), highly selective, and greatly sensitive detection of H_2_S at room temperature [122]. The sensor, developed using a simple chemical method, was able to detect concentrations as low as 100 ppb and respond in six minutes. This increase in sensitivity is attributed to changes in the carrier density of graphene when exposed to H_2_S, enabling real-time detection through resistivity changes and positioning it as a potential option for ambient gas sensing. Fascinatingly, the γ-Fe_2_O_3_ derived from MOF, dispersed on reduced graphene oxide (rGO), exhibited ultra-zero sensitivity for H_2_S at room temperature, displaying an impressive responsiveness of 520.73 to 97 ppm [123]. This sensor showed minimal cross-sensitivity to other gases and operated through engineered surface-controlled processes enhanced by the abundant active sites on rGO. The combination of the bulk resistance of γ-Fe_2_O_3_ and the conductivity of rGO facilitated low-power, high-performance H_2_S sensing suitable for environmental monitoring and industrial safety. Intriguingly, the MoS_2_ nanoparticle hybrids intercalated with GO sheets demonstrated quick and selective detection of H_2_S at room temperature [124]. The MoS_2_-GO hybrid sensor, containing 3 wt.%, showed a 39.16 percent response to 100 ppm of H_2_S, achieving a detection limit of 1 ppm and a swift response time of 6.13 s. The combined effects of MoS_2_ and GO improved gas adsorption and electron transport. Impedance spectroscopy indicated effective interaction mechanisms with H_2_S, highlighting its potential for real-time environmental applications. Captivatingly, MoO_3_ nanorods paired with rGO created hybrids that demonstrated enhanced H_2_S sensing capabilities at 110 °C [125]. These hybrids demonstrated greater sensitivity, faster rise and recovery times, and superior selectivity compared to pure MoO_3_. The formation of heterojunctions enhanced electron mobility, increasing the n-type character of the surface and improving conductivity. This hybridization strategy addresses the low conductivity issue of MoO_3_, presenting a promising approach for real gas sensors operating at moderate temperatures. Furthermore, Balasubramani et al. present an H_2_S gas sensor utilizing impedance spectroscopy, developed with reduced graphene oxide (rGO) combined with nano-ZnO (n-ZnO) composites [126]. The sensor exhibited optimal performance at 90 °C for H_2_S concentrations ranging from 2 to 100 ppm. The sensitivity of the rGO/n-ZnO composites significantly surpassed that of pure n-ZnO, owing to the excellent electrical conductivity of rGO and its increased adsorption sites. The grain boundary resistance played a critical role, decreasing as the H_2_S concentration increased, thereby facilitating gas detection. The sensor demonstrated rapid response (8 s) and recovery (32 s) times, notable selectivity towards H_2_S compared to other gases, along with great potential for real-time applications in environmental monitoring.

**Figure 6:**
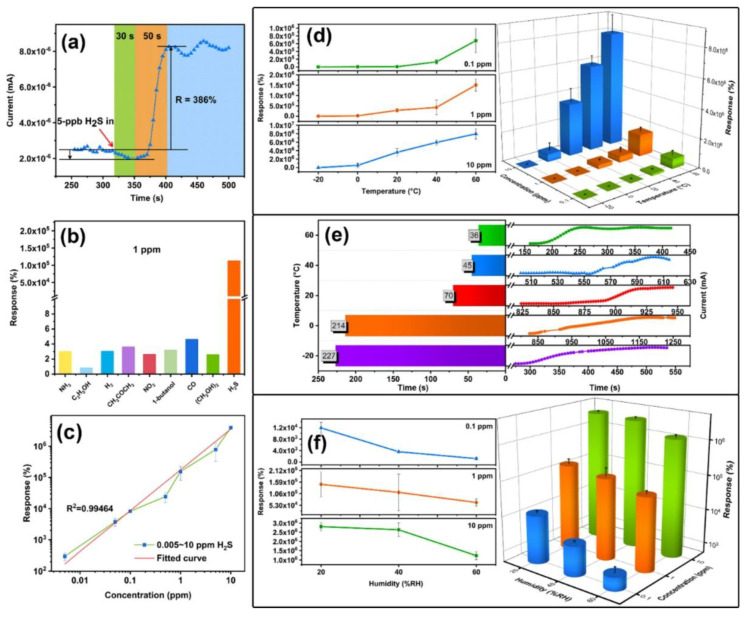
Gas-sensing performance of the Cu_2_O/graphene heterojunction sensor. (**a**) Dynamic response to 5 ppb H_2_S at 20 °C, (**b**) Selectivity toward 1 ppm H_2_S compared with interfering gases, (**c**) Response versus H_2_S concentration (5 ppb to 10 ppm), (**d**) Response at different operating temperatures (−20 °C to 60 °C), (**e**) Response time variation with temperature, and (**f**) Sensor responses under different relative humidity levels (20–60% RH). Reproduced with permission from Ref. [127]. Copyright (2025) Elsevier.

With advancements in the sensing capabilities of rGO-based nanocomposites, researchers are now exploring another avenue to enhance sensitivity and minimize detection limits through heterostructure engineering. By integrating p-type and n-type semiconductor materials into a hybrid structure, which not only promotes efficient charge transfer but also enables effective modulation of carrier concentration at the interface. This leads to improved sensitivity and selectivity in gas detection under ambient conditions, likely due to limitations imposed by high temperatures and power consumption. These advancements mark a shift toward sensors that are operable at room temperature, with improved performance metrics, applicable in biomedical and environmental sensing. A noteworthy outcome of the advancements of Cu_2_O/graphene heterojunction is illustrated in Figure 6a–f, which demonstrates the sensitivity of a newly developed sensor to extremely low concentrations of H_2_S approximately 5 ppb at room temperature (25 °C) [127]. The Cu_2_O/graphene heterojunction sensor exhibits a rapid increase in its response, showing a 386% response in merely 50 s. This study presents a highly sensitive and selective H_2_S gas sensor based on a Cu_2_O/graphene heterojunction. The Cu_2_O/graphene heterojunction sensor shows outstanding sensitivity at room temperature, capable of detecting H_2_S concentrations as low as 5 ppb, with a response rate of 386%. It also exhibits remarkable selectivity, responding to H_2_S much more strongly than to other common interference gases, such as NH_3_, NO_2_, and ethanol. The sensor’s dynamic response is rapid, with both a reaction time and recovery rate that improve as the temperature rises, from 214 s at 0 °C to 36 s at 60 °C. Additionally, it maintains stability under harsh conditions, including temperatures as low as −20 °C and high humidity levels of 60% relative humidity (RH), highlighting its durability. As a result, the Cu_2_O/graphene heterojunction sensor is highly effective at detecting minimal concentrations of biomarkers in a person’s breath. The mechanism involves both barrier modulation at the heterojunction and surface chemical reactions with H_2_S, along with switch-like behavior triggered by the presence of Cu_x_S at higher concentrations. Graphene’s high carrier mobility and large surface area enhance charge transport and gas adsorption. Support for quantitative detection can be derived from a linear relationship between log(response) and log(concentration). Additionally, a real-time exhaled breath analyzer, a portable prototype, was introduced, indicating its potential use in clinical diagnostics and environmental monitoring.

### 4.5. Humidity Sensing

Humidity sensing plays a vital role across various applications, including environmental monitoring, industrial automation, precision agriculture, healthcare, and consumer electronics. The safety, comfort, and proper functioning of devices and systems rely on accurate and efficient detection of airborne moisture. Traditional humidity sensors, such as resistive, capacitive, and optical sensors, face compatibility issues, including slow response times, hysteresis, drift, and poor long-term stability, especially in fluctuating conditions. To address these challenges, recent research has explored the incorporation of graphene and its derivatives, leveraging their exceptional electrical, structural, and chemical properties. Among the most promising advancements is the use of laser-induced graphene (LIG) on ethanol-treated polyimide (PI) films [128]. This approach enhances sensor performance by increasing the porosity and hydrophilicity of the PI film, which significantly boosts water molecule adsorption and ion conduction. Compared to untreated films, the ethanol-soaked PI sensors exhibited superior sensitivity (0.65%/RH), reduced hysteresis (5.1% RH), and extended detection distance to 30 cm for breath monitoring. Integration into sensor arrays further improved detection sensitivity and range, enabling fully contactless respiratory monitoring applications in both medical and athletic applications. Furthermore, graphene oxide (GO), when combined with electrospun poly(vinyl alcohol) (PVA) nanofibers, forms another potent sensor platform [129]. PVA nanofiber scaffolds were produced via electrospinning, which improved the electrical conductivity and surface interactions of the GO films. The optimized sensor demonstrated an impressive response rate of 98.44% at 80% relative humidity (RH) and exhibited remarkable stability over one week. The composite structure significantly enhanced water molecule adsorption and ion conduction. Furthermore, the sensors effectively identified human breathing patterns under various conditions (normal, deep, intermittent, coughing, and speech), highlighting their potential for medical diagnostics and wearable health monitoring applications. Additionally, the influence of graphene layer count and sensing area on sensor performance has also been systematically explored. This study investigates the effect of the number of atomic layers and the sensing area on graphene-based humidity sensors fabricated on SiO_2_/Si substrates [130]. Monolayer graphene devices exhibited the highest responsivity due to their enhanced interaction with the substrate. In contrast, tri-layer graphene achieved the fastest response and recovery times. Double-layer graphene offers a compromise between stability and sensitivity, making it an ideal material for applications such as respiratory monitoring and non-contact finger tracking. The findings indicated that responsivity decreases with an increased number of layers and a reduced sensing area, highlighting the significance of surface interactions and available adsorption sites. This study enhances our understanding of graphene’s humidity sensing properties in relation to layer count, thereby supporting its application in compact wearable sensors. On the other hand, combining reduced graphene oxide (rGO) with MoS_2_ led to further enhancements in sensitivity and flexibility [131]. A flexible humidity sensor utilizing a rGO/MoS_2_ composite was developed, exhibiting a rapid response time of 0.65 s and a remarkable responsivity of 96.7% over a relative humidity (RH) range of 11–95%. The combination of rGO’s large surface area and MoS_2_’s hydrophilic properties enabled excellent absorption of water molecules and quick electron transfer. The sensor maintained stability under bending conditions, exhibited favorable hysteresis behavior, and provided a linear response across a wide relative humidity (RH) range. When combined with flexible circuits, it effectively tracked human breath and served as a non-contact switch, showcasing its potential uses in wearable technology and real-time environmental monitoring. Interestingly, a self-powered capacitive humidity sensor was developed using graphene oxide (GO) and GO intercalated with poly(sodium 4-styrenesulfonate) (PSS) [132]. Adding PSS increases water permeation by expanding the interlayer spacing, which improves sensitivity. The GO-PSS sensors exhibited approximately 5.6 times higher voltage output and three times faster response times compared to GO sensors across a 0–80% relative humidity range. Capacitance changes were attributed to water adsorption and sodium ion mobility, boosting the electrical double-layer capacitance. The sensor generated voltage in a charge-pumping setup, highlighting its capacity for humidity-based energy harvesting. Moreover, an attractive study shows that electron irradiation of a graphene/polymer composite humidity sensor boosts its efficiency [133]. Under optimal irradiation, the sensor’s responsiveness increased by a factor of 22.4, achieving a performance level of 1.2 × 10^5^ at 97.3% relative humidity. The process created surface defects, allowed functional groups to interact with oxygen gas, and increased anti-crosslinking density, which improved the composite’s surface area and water absorption. Additionally, the sensor demonstrated faster response and recovery times, along with enhanced bending stability and water resistance, highlighting its suitability for wearable and non-contact sensing applications. Therefore, these results indicate that electron irradiation is an effective post-processing technique for developing high-performance humidity sensors. Additionally, a simple and affordable method for producing ultrasonically responsive, fast-reacting flexible humidity sensors based on laser-induced graphene (LIG) on polyimide has been developed [134]. The sensor demonstrated exceptional sensitivity, with a 28,231-fold increase in current as the relative humidity increased from 26.1% to 90.2%. It showed quick response and recovery times of 0.5 s and 0.7 s, respectively. The ionic conduction of the polyimide layer, due to its high porosity and hydrophilicity, enabled performance that surpassed that of commercial MEMS sensors. Its practical applications included real-time respiration monitoring, grain moisture measurement, and water seepage detection, demonstrating the sensor’s potential for widespread use. Anichini et al. demonstrated that highly sensitive and ultrafast humidity sensors made from reduced graphene oxide (rGO) with chemical functionalization are highly versatile [135]. By functionalizing with triethylene glycol chains, they developed rCMGO-1, which showed the best performance, with 31% higher resistance across a 2% to 97% relative humidity (RH) range, a response time of 25 ms, and a recovery time of 127 ms. This outperformed both unfunctionalized rGO and alkyl-functionalized rGO (rCMGO-2). rCMGO-1 exhibited low hysteresis, excellent repeatability, and strong moisture selectivity for organic volatiles. The enhanced sensing capability is attributed to supramolecular interactions between water molecules and hydrophilic functional groups, confirmed through XRD and molecular dynamics simulations. Interestingly, a 2D MoS_2_-graphene nanocomposite was used to develop a new humidity sensor that can be mass-produced and operates independently of temperature [136]. This sensor was produced using an all-printed laser ablation method, where laser-induced graphene (LIG) acts as the transducer electrodes, and MoS_2_/graphene forms the base layer. It offers high performance, covering a 0 to 100% RH detection range, with quick response and recovery times (4 s/2 s), and maintains high stability across various temperatures due to the competing thermal responses of MoS_2_ and graphene. The sensor exhibits minimal hysteresis (3.94%) and excellent reproducibility (1.9% error). Notably, a high-performance humidity sensor is improved with ultrathin graphene oxide (GO) films that are doped during vacuum annealing [137]. The sensor enables real-time applications due to its ultra-fast response and recovery times (0.32 s and 0.42 s), allowing for breath and proximity detection, among other uses. On flexible substrates, the sensor also demonstrated good reproducibility, supporting its potential for wearable health monitoring devices. These results highlight the sensor’s higher sensitivity, rapid, and stable performance in next-generation humidity measurements. Remarkably, a new resistive humidity sensor has been developed using graphene oxide (GO), lithium-doped (Li-GO), and boron-doped (B-GO) [138]. Li-GO has been found to have outstanding gas sensing properties, showing excellent sensitivity (3038.26%), a rapid response (4 s), and recovery (25 s) over a wide humidity range (11 to 97% RH). This outperformed undoped GO and B-GO, likely due to the increased interlayer spacing and hydrophilic LiOH functional groups, which enhanced water molecule adsorption and improved charge transport. The sensor demonstrated excellent linearity, minimal hysteresis (0.83%), and stable performance over time. Additionally, it is highly cost-effective and efficient for monitoring ambient relative humidity.

**Figure 7:**
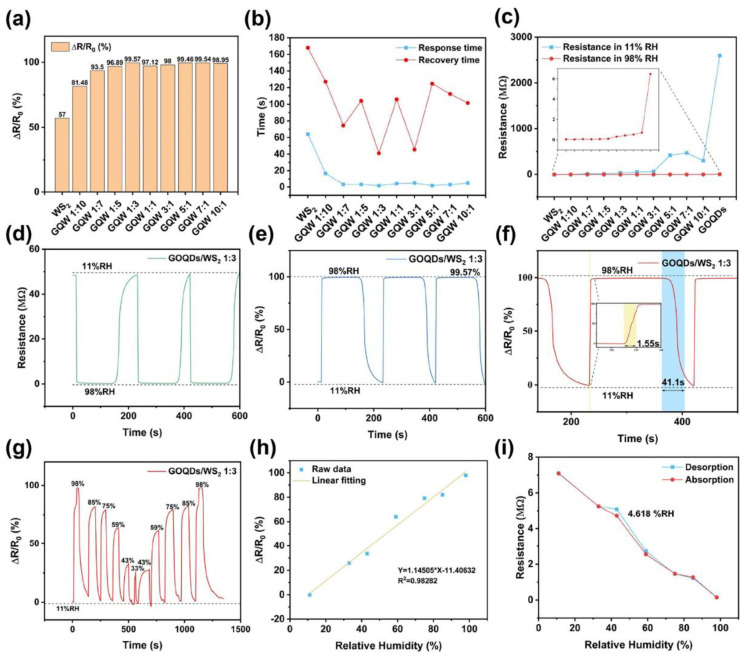
Comparison between the performance of flexible sensors based on pure WS_2_ and GOQDs/WS_2_ composite in terms of humidity sensing. (**a**,**b**) Responsivity, response time and recovery time across various ratios, (**c**) values of resistance at 11% and 98% RH, (**d**) repeatability test of GOQDs/WS_2_ (1:3), (**e**) responsivity 99.57%, (**f**) response time of 1.55 s and recovery time of 4.11 s, (**g**) stepwise RH sensing, (**h**) linear correlation between responsivity and RH, and (**i**) relation between resistance and relative humidity. Reproduced with permission from Ref. [139]. Copyright (2025) American Chemical Society.

Figure 7a–i shows a comparison of the humidity sensing ability of GOQDs/WS_2_-based hybrid sensors of different GOQDs mixing ratios and the optimization and better performance compared to pure WS_2_ sensor [139]. Figure 7a,b shows responsivity, response time, and recovery time of various GOQDs and WS_2_ ratios. Notably, the highest responsivity (99.57%), fastest response (1.55 s), and shortest recovery time (41.1 s) are observed in the GOQDs/WS_2_ (1:3 ratio) composite. This remarkable improvement, compared to the bare WS_2_ sensor (57% responsivity, 64.2 s response time, and 168 s recovery time), again demonstrates the synergistic effect of GOQDs in the GOQDs/WS_2_-based hybrid sensor. The GOQDs provide abundant oxygen-containing functional groups and a large surface area, which enhances water molecule adsorption and promotes proton conduction. The resistance of sensors at 11% and 98% RH is indicated at different compositions, as depicted in Figure 7c. With increasing the GOQDs amount, the resistance increases as a result of the insulating nature of GOQDs. Nevertheless, the composite has retained a high sensitivity, indicating that it exhibits an enhanced balance of conductivity and water interaction. Figure 7d clearly demonstrates the repeatability and stability of the sensor responses. The consistent behavior of the GOQDs/WS_2_ (1:3) sensor was also evidenced by only minor signal variations over 100 cycles at high and low humidity levels. Additionally, Figure 7e reaffirmed the sensor’s high responsivity at 98% RH, while Figure 7f detailed the quick and smooth transition during the response and recovery processes. These features are essential for practical real-time monitoring applications. Figure 7g illustrates a humidity step-response experiment where the sensor’s resistance fluctuates as RH increases. The consistent and distinct signals at different humidity levels highlight the sensor’s potential for accurate, continuous RH monitoring. Notably, the linear correlation shown in Figure 7h confirms that sensor responsivity is strongly linked to relative humidity, enabling precise calibration and data analysis. Additionally, Figure 7i reveals low hysteresis, with a maximum of just 4.618% RH at 43% RH, indicating the device’s reliability and minimal memory effect, essential qualities for long-term stability in wearable applications.

## 5. Challenges and Opportunities

Despite considerable advancements, the broad application of graphene-based gas sensors still faces several significant challenges. Addressing these problems while seizing emerging opportunities is crucial for unlocking the full potential of graphene in real-world sensing technologies.

### 5.1. Long-Term Stability and Robustness

A key challenge is ensuring that sensor performance remains stable over time under different environmental conditions. Variables such as humidity, temperature changes, and prolonged exposure to reactive gases can harm functional groups, dopants, or the graphene structure itself. To tackle this, strategies include: creating protective yet breathable coatings to shield graphene without blocking gas access; engineering stable functionalization methods that withstand environmental degradation; and integrating self-healing or reconfigurable materials into the sensor design. Enhancing robustness is crucial for use in outdoor, industrial, and biomedical settings where conditions are less controlled.

### 5.2. Large-Scale and Cost-Effective Fabrication

While laboratory-scale production of high-quality graphene is well-developed, mass production that maintains consistency is still a significant challenge. Issues include the following: producing uniform monolayer or few-layer graphene over large surfaces; managing defect density and layer stacking during chemical vapor deposition (CVD); and creating reliable, scalable functionalization and device fabrication methods. Progress in roll-to-roll manufacturing, inkjet printing of graphene inks, and scalable transfer techniques are promising steps toward making graphene-based sensors commercially feasible.

### 5.3. Enhancing Selectivity

Selectivity remains a significant barrier for practical applications. The absence of inherent specificity in pristine graphene means it reacts to a broad spectrum of gases, complicating detection in mixed environments. Potential solutions include the following: strategically designing selective functional groups and molecular receptors; utilizing multi-functionalized sensor arrays in conjunction with machine learning for pattern recognition; and employing hybrid materials that selectively catalyze or adsorb specific gases [140]. Achieving tailored selectivity without compromising sensitivity or response time is vital for clinical diagnostics, environmental monitoring, and industrial safety.

## 6. Future Perspectives

The area of graphene-based gas sensors is advancing quickly, propelled by technological innovations and urgent societal demands for more intelligent, responsive sensing systems. In the future, several crucial directions are expected to influence the development of gas sensing research and its applications.

### 6.1. Next-Generation Sensor Arrays Combined with Graphene Derivatives

To further enhance sensor performance, research is increasingly focused on graphene derivatives, including graphene oxide (GO), reduced graphene oxide (rGO), fluorinated graphene, and nitrogen-doped graphene. These materials offer tunable surface chemistry, defect densities, and electronic properties that can be tailored for specific gas sensing tasks. Additionally, hybrid systems combining graphene with other two-dimensional (2D) materials, such as transition metal dichalcogenides (MoS_2_ and WS_2_), MXenes, or black phosphorus, are emerging as powerful platforms for creating multifunctional, highly selective sensing devices. Designing 2D/2D heterostructures with engineered band alignments and interfacial charge transfer dynamics holds great promise for achieving breakthroughs in sensitivity and selectivity. Interestingly, next-generation sensor arrays integrated with graphene derivatives can create electronic nose architectures that interpret complex gas signatures via pattern recognition, rather than relying on the selectivity of individual sensors.

### 6.2. Multi-Analyte Sensing and Selective Recognition

Graphene-based sensors are anticipated to advance from detecting single gases to identifying multiple substances simultaneously [141]. This shift will require creating arrays of sensors that are selectively functionalized to recognize and differentiate various gases at once. It will also involve integrating data fusion and pattern recognition algorithms to analyze complex sensor signals. Designing materials and device structures that produce distinct or complementary responses to different analytes will be crucial. These improvements are vital for applications such as medical diagnostics, environmental pollution monitoring, and industrial process control. Recent research suggests exploring ultrathin water layers on semiconductor surfaces that can dynamically adsorb and dissociate gases, forming acids or bases and causing local pH changes. These interactions can be detected electronically and reversed via desorption, enabling reversible and selective sensing. With their large surface area, high conductivity, and compatibility with aqueous environments, graphene sensors are excellent platforms for studying these effects and developing reusable sensors.

### 6.3. Towards Real-World Deployment: Flexible, Wearable, and Self-Powered Sensors

Integrating graphene sensors into flexible, stretchable, and wearable platforms marks a pivotal trend in practical applications. The advancements in flexible electronics, along with graphene’s durability and lightweight properties, facilitate the design of sensors that adapt to uneven surfaces and maintain reliability under mechanical stress. Additionally, there is increasing interest in producing self-powered gas sensors using energy-harvesting technologies like triboelectric nanogenerators and solar components, enabling functionality without external power sources. These developments are crucial for creating genuinely portable and decentralized sensing networks.

### 6.4. Commercialization Challenges

To ensure broad market acceptance of graphene-based sensors, it is essential to address regulatory and standardization hurdles. This includes establishing standardized testing methods for sensor performance in real-world conditions, ensuring reproducibility and consistency across production batches, and adhering to safety and environmental regulations related to graphene materials and device components. Collaborations among academia, industry, and regulatory agencies will play a crucial role in accelerating the transition of graphene gas sensing technologies from research environments to commercial markets. Besides the technical challenges mentioned earlier, it is essential to focus on the practical needs of industrial gas sensors. Industrial use demands a balance of good sensitivity, quick response, stability during operation, affordability, and straightforward deployment. While academic research often aims for ultra-low detection limits and high selectivity, many real-world uses require detection in the ppm range, moderate selectivity, and stable performance over several months. Additionally, ease of manufacturing and system integration are vital, and pursuing extremely high-performance goals may not always be necessary or realistic. Consequently, sensor development should align with the practical requirements of specific applications to facilitate a smoother shift from lab research to commercial use.

### 6.5. Innovative Pathways Forward

To address the ongoing challenge of limited selectivity in graphene-based gas sensors where multiple analytes influence a single electrical signal, new strategies are emerging that emphasize multimodal sensing and data-driven techniques. A practical method involves dual-parameter measurements, such as combining electrical resistance with optical or acoustic wave signals. These independent outputs provide a more detailed fingerprint of the analyte-sensor interaction, enhancing accuracy in complex mixtures. Moreover, machine learning algorithms are increasingly employed to distinguish overlapping signals from multiplexed sensor arrays, boosting classification and prediction accuracy [142,143]. Combining graphene with two-dimensional heterostructures or metal–organic frameworks also allows for tailored chemical interactions and electronic modifications, leading to highly selective gas detection. These hybrid structures improve sensor specificity and performance under ambient conditions. Overall, these advances reflect a move toward intelligent, multi-signal, material-engineered gas sensing systems capable of meeting real-world selectivity needs.

## 7. Conclusions

Graphene-based gas sensors are viewed as up-and-coming candidates for the next generation of chemical sensing, due to their exceptional surface area, remarkable electrical properties, and the potential for precise modifications through functionalization and hybridization. In the last decade, significant strides have been made in comprehending the core sensing mechanisms, such as charge transfer, Fermi level shifts, and quantum interactions within nanoscale structures. Concurrently, advances in both covalent and non-covalent functionalization, doping, defect engineering, and the integration of hybrid materials have created new opportunities to improve sensor sensitivity, selectivity, stability, and response dynamics. The practical application of graphene-based gas sensors is swiftly broadening, covering essential areas like environmental monitoring, medical diagnostics, and industrial safety. However, considerable challenges regarding long-term stability, large-scale manufacturing, and selective detection still impede widespread commercialization. Emerging trends, including machine learning-enhanced sensing, compatibility with flexible and wearable devices, and the development of multi-analyte sensing arrays, offer significant potential for overcoming these obstacles. Looking forward, the future of graphene gas sensors hinges on interdisciplinary innovation that combines materials science, engineering, data analytics, and system integration. By tackling existing challenges and seizing new technological opportunities, graphene-based gas sensing platforms are set to become integral to the development of smarter, safer, and healthier societies.
